# Transition From Psoriasis to Psoriatic Arthritis is Characterized by Distinct Alterations in Peripheral Blood Tc17, Th17, and CD4 ^+^ Effector Memory Cells

**DOI:** 10.1002/art.43396

**Published:** 2025-12-28

**Authors:** Hanna Graßhoff, Sara Comdühr, Henry Nording, Jacob von Esebeck, Philine Letz, Miriam Prohaczka, Handan Gedik, Henner Zirpel, Elisabeth Spallek, Linh Ha‐Wissel, El‐Baraa Adjailia, Sabrina Arnold, Konstantinos Fourlakis, Sebastian Klapa, Ingo Eitel, Oliver J. Müller, Jens Y. Humrich, Gabriela Riemekasten, Diamant Thaçi, Peter Lamprecht

**Affiliations:** ^1^ Department of Rheumatology and Clinical Immunology University of Lübeck Lübeck Germany; ^2^ Cardioimmunology Group, Medical Clinic II University Heart Center Lübeck Lübeck Germany; ^3^ Department of Internal Medicine V University of Kiel Kiel Germany; ^4^ German Centre for Cardiovascular Research Partner Site Hamburg/Kiel/Lübeck Lübeck Germany; ^5^ I. Department of Medicine University Medical Center Hamburg‐Eppendorf Hamburg Germany; ^6^ Hamburg Center for Translational Immunology University Medical Center Hamburg‐Eppendorf Hamburg Germany; ^7^ Institute and Comprehensive Center for Inflammation Medicine University of Lübeck Lübeck Germany; ^8^ University Heart Center Lübeck, Medical Clinic II, University of Lübeck Lübeck Germany

## Abstract

**Objective:**

Cellular mechanisms driving transition from psoriasis to psoriatic arthritis have remained largely elusive. Thus, we investigated changes within the peripheral blood T cell compartment associated with the transition phase.

**Methods:**

In an observational study, 116 patients were examined and categorized into subgroups including psoriasis with at least one risk factor for transition to psoriatic arthritis, subclinical psoriatic arthritis according to EULAR taskforce recommendations from 2023, and definitive psoriatic arthritis meeting the Classification Criteria for Psoriatic Arthritis. Demographic and clinical characteristics of patient subgroups were analyzed. Deep T cell phenotyping using multicolor flow cytometry and machine‐learning techniques were applied.

**Results:**

Overlapping T cell endotypes were found among patients with subclinical psoriatic arthritis exhibiting the most notable divergence from the others. Frequencies of effector memory CD4^+^ T cells, Th17, and Tc17 cells differed among patients with psoriasis with at least one risk factor for transition, subclinical psoriatic arthritis, and psoriatic arthritis. Transition‐associated changes of Tc17 cell frequencies were confirmed by machine‐learning–assisted unsupervised clustering analysis. Moreover, patients with enthesitis could be distinguished from those without, with Tc17 cells being the main distinctive feature.

**Conclusion:**

Transition from psoriasis to psoriatic arthritis was associated with distinct alterations of the peripheral blood T cell compartment, with Tc17 cells exhibiting the greatest discriminatory power. These findings provide insight into pathomechanisms driving disease progression during transition from psoriasis to psoriatic arthritis and identify Tc17 cells as the foremost novel potential therapeutic target for the prevention of transition.

## INTRODUCTION

Psoriasis is a chronic systemic immune‐mediated disease characterized by T cell–driven epidermal hyperplasia with a prevalence between 0.51% and 11.43% in adults depending on ethnic and geographic variation.^1^ In up to 30% of patients, the disease is associated with the development of psoriatic arthritis characterized by synovioentheseal inflammation.^2^ Psoriasis precedes psoriatic arthritis in about 70% of the patients by an average of seven to eight years with an annual transition rate of 0.74% and 2.7%.^3^, ^4^, ^5^ Risk factors for transition to psoriatic arthritis include younger age at disease onset, psoriasis severity, phenotype (nail, scalp, and inverse psoriasis), comorbidities (obesity), environmental factors (infections, smoking, trauma, emotional stress, medications), and genetic factors.^6^, ^7^, ^8^, ^9^ Although these risk factors are associated with an increased rate of transition, they do not necessarily indicate an imminent progression to psoriatic arthritis. Instead, they are considered components within a complex, multifactorial landscape that underlies disease progression.

Although T cells are known to be the key drivers of pathophysiology in psoriasis and psoriatic arthritis, current knowledge regarding immunologic processes during disease transition remains scarce. Evidence for CD8^+^ T cell involvement in the pathogenesis comes from the strong association with major histocompatibility complex (MHC) class I genes, notably HLA‐C*0602, and single nucleotide polymorphisms of the interleukin (IL)‐23 and IL‐23 receptor (IL‐23R) genes promoting Th17 cell differentiation.^8^, ^9^ Increased frequencies of circulating Th1, Th17, and Th22 CD4^+^ T helper cell subsets have been reported in psoriasis as well as enrichment of Th1, Th9, Th17, and Th22 CD4^+^ T cells and their CD8^+^ cytotoxic counterparts (Tc1, Tc17, and Tc22) in psoriatic lesions.^10^, ^11^, ^12^, ^13^, ^14^, ^15^, ^16^ Unlike psoriasis, immunopathogenic features of T cells are less well characterized in psoriatic arthritis. There is a gap in knowledge of the pathogenic relationship between T cell responses in psoriasis and its link to the development of joint inflammation.^17^ It remains unclear which changes in T cell populations occur during transition from psoriasis to psoriatic arthritis and in which way T cells contribute to this transition.

Biologic agents targeting tumor necrosis factor (TNF) α, IL‐17, and IL‐23 have improved the outcome of psoriatic arthritis, albeit with a rate of 30% to 40% nonresponders or inadequate responders. By contrast, improvement of skin lesions in psoriasis with biologics, particularly those that inhibit the IL‐23/IL‐17 axis, is far more effective than the joint response to these biologics.^18^, ^19^ Notably, unlike Th17 cells, Tc17 cells lack therapeutic responsiveness and are retained in resolved psoriatic skin lesions, thus potentially driving recurrent inflammation and facilitating transition from psoriasis to psoriatic arthritis.^20^ A promising strategy to prevent sequelae and improve patient outcomes is therefore the early identification and targeted treatment of individuals at risk of developing psoriatic arthritis.^8^ Clinical studies support the concept of a “window of opportunity,” during which intervention can alter the progression to psoriatic arthritis, and they indicate that early treatment leads to better long‐term outcomes.^8^, ^21^ This highlights a significant unmet medical need, namely a deeper understanding of the immunopathological mechanisms underlying the transition phase, and more precise identification of patients with psoriasis at increased risk of developing psoriatic arthritis.^8^ To facilitate comparability across studies, an EULAR task force established classification criteria for patients in transitional stages. As an intermediate group bridging psoriasis and early psoriatic arthritis, they identified a category termed “subclinical psoriatic arthritis,” characterized by arthralgia and/or imaging evidence of synovioentheseal inflammation.^22^, ^23^ According to Zabotti et al, the probability for fulfilling the Classification of Psoriatic Arthritis (CASPAR) criteria among patients with subclinical psoriatic arthritis who report arthralgia is 22.7% after 36 months.^24^ This observation emphasizes the importance of recognizing musculoskeletal symptoms for identification of patients undergoing transition early.

In this study, we hypothesized that the transition phase is characterized by changes in the composition of T cell subsets distinct from psoriasis and psoriatic arthritis, assuming that immune dysregulation precedes clinically apparent joint inflammation. To identify changes within the peripheral blood T cell compartment and clinical features associated with the transition from psoriasis to psoriatic arthritis, we studied 116 patients subgrouped into psoriasis with risk factors for psoriatic arthritis, subclinical psoriatic arthritis, and psoriatic arthritis. Demographic and clinical manifestations of psoriasis and psoriatic arthritis were assessed, and deep T cell phenotyping was performed.

## MATERIALS AND METHODS

Detailed experimental procedures are provided in the Supplemental Materials. Consecutive patients with psoriasis (n = 46), in the transition phase (n = 8), and with psoriatic arthritis (n = 62) were included. Due to the exploratory nature of the study, no formal sample size calculation was performed, and patient numbers were based on feasibility and the aim to capture relevant clinical diversity. The diagnosis of psoriasis and nail involvement was made clinically by a dermatologist. Inclusion criteria for patients with psoriasis were (i) at least one risk factor for transition to psoriatic arthritis including obesity, severe psoriasis, nail involvement, and a first‐degree relative with psoriatic arthritis, and (ii) active disease with an indication for initiation of systemic therapy according to the 2021 S3 guideline for the treatment of psoriasis.^25^ The group of patients with psoriasis with at least one risk factor for transition to psoriatic arthritis is termed “psoriasis” in the following. Subclinical psoriatic arthritis was defined as psoriasis with arthralgia and/or imaging evidence of synovial/entheseal inflammation without clinical synovitis, as suggested by an EULAR taskforce in 2023.^23^ Psoriatic arthritis was diagnosed by rheumatologists following the CASPAR criteria.^26^ Additionally, these patients had an active disease with an indication for initiation of systemic therapy in accordance with current EULAR and Group for Research and Assessment of Psoriasis and Psoriatic Arthritis (GRAPPA) recommendations.^27^, ^28^ Individuals who were pregnant or actively lactating were excluded from study participation.

Demographic parameters, clinical data, and laboratory parameters were assessed. Disease activity was scored using Psoriasis Area Severity Index (PASI), Dermatology Life Quality Index (DLQI), itch on a visual analog scale (VAS), tender and swollen joint count according to Disease Activity in Psoriatic Arthritis score, German Psoriasis Arthritis Diagnostic questionnaire, Leeds Enthesitis Index and the Maastricht Ankylosing Spondylitis Enthesitis as well as Health Assessment Questionnaire (HAQ). Details of the diagnostic pathway to classify study participants are shown in Supplementary Figure 1.

Deep T cell phenotyping was conducted in all patients using multicolor flow cytometry on heparinized blood samples, following standardized staining, lysis, and viability protocols. The samples were acquired using CytoFLEX S spectral analyzer flow cytometer (Beckman Coulter) and CytExpert software (Beckman Coulter). Data analysis was performed using FlowJo version 10 (BD Biosciences). The gating strategy is presented in Supplementary Table 1, Supplementary Figures 2 and 3. Influence of age, sex and BMI of peripheral T cells is shown in Supplementary Figure 4. To assess therapeutic effects on T cell subset frequencies, 114 patients underwent immunophenotyping at therapy initiation and after 2, 4, and 16 weeks.

The statistical analysis to identify T cell subsets important in transition from psoriasis to psoriatic arthritis was performed using the software R version 4.3.3 und GraphPad Prism version 10.0 (GraphPad Software, Inc). These analyses included Partial Least Squares–Discriminant Analysis (PLS‐DA), frequency comparison of T cell subsets using Kruskal–Wallis test, and Random Forest analyses. The dataset for Random Forest analyses was prepared by imputing missing values with k‐nearest neighbors.

To confirm the importance of identified T cell subsets independent of sex, age, and body mass index (BMI), we performed a machine‐learning (ML)‐based flow cytometry data processing in R statistical environment (version 4.2). All events from a subcohort matched by age, sex, and BMI were combined into one flow set, gated on live CD3^+^ lymphocytes. After preprocessing of the data by compensating for spectral overlap, gating for singlets and live cells and applying quality control, the data were transformed and scaled. Thereafter, we performed Gaussian normalization to mitigate batch effects. We then employed the self‐organizing map algorithm FlowSOM to perform unsupervised clustering of cells based on the expression states of selected markers. Uniform manifold approximation and projection (UMAP) plots were generated using type markers, with a limit of 20,000 cells per sample. We conducted a differential abundance analysis to compare cluster numbers.

Ethical approval was obtained from local ethics committee (AZ20‐170A). The datasets generated and analyzed during the current study are available from the corresponding author on reasonable request. Requests for data access should be directed to Dr Graßhoff at hanna.grasshoff@uksh.de. Patients or the public were not involved in the design, conduct, reporting, or dissemination plans of our research.

## RESULTS

### Patient demographics and disease subgroups

In this study, 116 consecutive patients were included and observed at the Institute and Comprehensive Center for Inflammation Medicine and Department of Rheumatology and Clinical Immunology at the University of Lübeck. The cohort was composed of patients with psoriasis (n = 46, 39.7%), with subclinical psoriatic arthritis (n = 8, 6.9%), and with psoriatic arthritis (n = 62, 53.4%). Patient demographics and clinical data are summarized in Table 1 and Supplementary Table 2. Patients with psoriatic arthritis tended to be older than patients with psoriasis and subclinical psoriatic arthritis. Sex ratio and BMI were similar among disease groups. Patients with psoriasis showed significantly higher PASI and body surface area scores, indicating more severe skin involvement compared with subclinical psoriatic arthritis and definitive psoriatic arthritis. Moreover, DLQI scoring and mean itch (VAS) were significantly higher in patients with psoriasis, indicating a higher skin disease burden. By contrast, nail psoriasis was more frequent in patients with subclinical psoriatic arthritis and psoriatic arthritis. Pain assessed via VAS and scoring in HAQ—both assessing subjective disease burden mainly caused by musculoskeletal manifestations—was similar in patients with subclinical psoriatic arthritis and those with definitive psoriatic arthritis. In two patients in the subclinical psoriatic arthritis group, a swollen joint was detected caused by trauma and after surgery. Yet, none of the patients in the subclinical psoriatic arthritis group had clinically apparent arthritis, enthesitis, axial disease manifestation, dactylitis, or uveitis. Patients were mainly treated with conventional synthetic disease‐modifying antirheumatic drugs (csDMARDs) and biologics.

**Table 1:** Clinic and demographic characterization of patients with psoriasis, subclinical psoriatic arthritis, and psoriatic arthritis included in the immunophenotyping cohort*

Parameters	Psoriasis (n = 46, 39.7%)	Subclinical psoriatic arthritis (n = 8, 6.9%)	Psoriatic arthritis (n = 62, 53.4%)	Statistical analysis
Demographics and Clinical Data	M (±SD)	M (±SD)	M (±SD)	Kruskal‐Wallis statistic: H(2) (P value)
Age, y	43.5 (±14.3)	42.0 (±14.1)	47.9 (±13.3)	4.08 (0.130)
BMI, kg/m^2^	32.1 (±7.8)	27.9 (±5.1)	30.5 (±5.5)	3.09 (0.213)
PASI, 0–72	15.3 (±8.9)	2.1 (±3.7)	5.7 (±6.2)	**45.0 (<0.001)**
BSA, %	21.4 (±16.6)	2.5 (±4.0)	6.7 (±7.7)	**43.1 (<0.001)**
DLQI, 0–30	12.8 (±8.4)	5.3 (±6.6)	8.5 (±7.1)	**10.3 (0.006)**
VAS itch mean, 0–10	5.8 (±3.0)	3.8 (±3.7)	4.3 (±3.4)	**0.023 (0.023)**
VAS pain mean, 0–10	3.5 (±3.4)	5.3 (±1.4)	5.9 (±2.7)	**0.001 (0.001)**
	n (%)	n (%)	n (%)	Fisher's exact test: P value
Female	15 (32.6)	5 (62.5)	31 (50.0)	0.106
Nail psoriasis	41 (89.1)	3 (37.5)	48 (77.4)	**0.006**
Therapy	M (±SD)	M (±SD)	M (±SD)	X^2^ test: X^2^ (P value)
Solely topical therapy	19 (41.3)	5 (62.5)	16 (25.8)	
csDMARD	6 (13.0)	1 (12.5)	20 (32.3)	
Fumarates	8 (17.4)	1 (12.5)	2 (3.3)	
Apremilast	2 (4.4)	1 (12.5)	4 (6.5)	
IL‐(12)/23 i	3 (6.5)	0 (0.0)	5 (8.1)	
TNF i	3 (6.5)	0 (0.0)	10^a^ (16.1^a^)	
IL‐17 i	5 (10.9)	0 (0.0)	4^a^ (6.5^a^)	
JAK i	0 (0.0)	0 (0.0)	1 (1.6)	20.9 (0.104)

* BMI, body mass index; BSA, body surface area; csDMARD, conventional synthetic disease‐modifying antirheumatic drug; DLQI, Dermatology Life Quality Index; i, inhibitor; IL, interleukin; PASI, Psoriasis Area Severity Index; TNF, tumor necrosis factor; VAS, visual analog scale.

^a^ One of the patients received biologics and methotrexate.

### Patients with psoriasis, subclinical psoriatic arthritis, and psoriatic arthritis display overlapping T cell subset endotypes with remarkable divergence in subclinical psoriatic arthritis

Multicolor flow cytometry was performed in all 116 patients. Total number of lymphocytes as well as frequencies of CD3^+^ lymphocytes were compared using Kruskal–Wallis test, indicating no statistically significant difference regarding absolute number of lymphocytes among the three patient subgroups [H (2) = 2.05, *P* = 0.358]. There were also no differences in CD3^+^ T cell frequencies among patient subgroups [H (2) = 4.09, *P* = 0.129, Kruskal–Wallis test].

**Figure 1:**
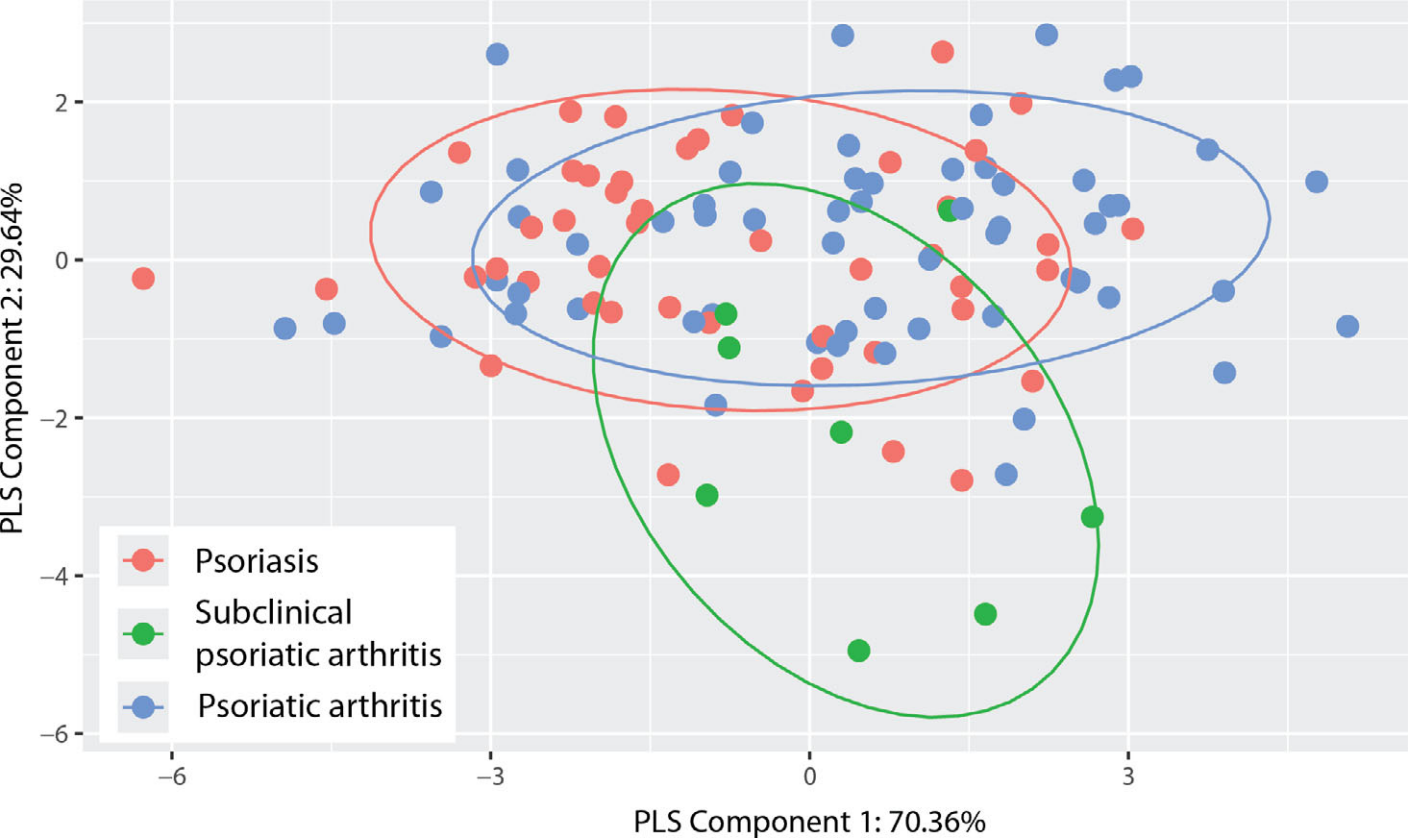
Patients with psoriasis, subclinical psoriatic arthritis, and psoriatic arthritis display overlapping T cell subset endotypes with remarkable divergence in subclinical psoriatic arthritis. Partial Least Squares (PLS)‐Discriminant Analysis showed circulating T cell endotypes shared among patients with psoriasis (red), patients with subclinical psoriatic arthritis (green), and patients with psoriatic arthritis (blue). Patients with subclinical psoriatic arthritis displayed the most remarkable divergence from the other two patient subgroups regarding endotypes. The results are illustrated as a PLS score plot with confidence ellipses set at 80%.

Figure 1 shows outcomes of the PLS‐DA analyzing the T cell composition suggesting the presence of overlapping peripheral blood T cell endotypes among the three patient subgroups. Nevertheless, patients with subclinical psoriatic arthritis displayed the most remarkable divergence from the other two patient subgroups regarding endotypes. Spearman's correlations among T cell subsets were calculated for each group separately and the interrelationships among the T cell subsets within each subgroup illustrated by correlation networks. Patients with subclinical psoriatic arthritis revealed a more intricate and interconnected T cell subset network compared to patients with psoriasis and psoriatic arthritis (Supplementary Figure 5).

### Frequencies of Th17, Tc17, and CD4 ^+^ effector memory T cells differ among patients with psoriasis, subclinical psoriatic arthritis, and psoriatic arthritis

**Figure 2:**
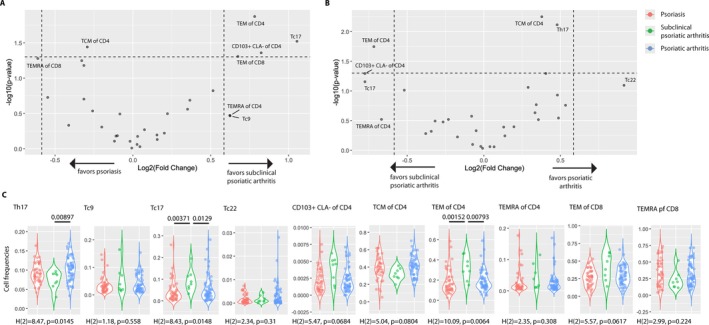
Frequencies of Th17, Tc17, and CD4^+^ TEM cells differ among patients with psoriasis, subclinical psoriatic arthritis, and psoriatic arthritis. Volcano plots are a visual representation of the results of the Student's *t*‐test for different peripheral blood T cell subset frequencies in patients with (A) psoriasis and subclinical psoriatic arthritis and (B) subclinical psoriatic arthritis and psoriatic arthritis. The *P* values are represented on the y‐axis as −log10(*P*), with a *P* value of 0.05 indicated by the dotted line. The fold change is illustrated as log2(fold change), and the arrows on the x‐axis indicate the direction of change. The dotted line represents a fold change >1.5. T cell subsets with a fold change of 1.5 were labeled. (C) Violin plots representing the distribution of T cell frequencies for the T cell subsets with a fold change >1.5 in patients with psoriasis, subclinical psoriatic arthritis, and psoriatic arthritis. The results of a Kruskal–Wallis analysis and subsequent Dunn's post hoc test are depicted in the respective plots. Significant *P* values in Dunn's post hoc test are indicated. CLA, cutaneous Ly; TCM, central memory T; TEM, effector memory T; TEMRA; TEM cells re‐expressing CD45RA.

To investigate the distribution of distinct T cell subsets, a comparison of the frequencies of different circulating T cell subsets was performed using Student's *t*‐test. The comparison between patients with psoriasis and subclinical psoriatic arthritis revealed a significant difference with a fold change >±1.5 for CD8^+^ effector memory T cells re‐expressing CD45RA (TEMRAs), CD4^+^ central memory T (TCM) cells, CD4^+^ effector memory T (TEM) cells, Tc17 cells, CD103^+^ cutaneous Ly (CLA)^−^ cells within the CD4^+^ T cell population, CD8^+^ TEM cells, CD4^+^ TEMRAs, and Tc9 cells (Figure 2A). A corresponding comparison of frequencies of T cell subsets between patients with subclinical psoriatic arthritis and psoriatic arthritis showed a significant difference with a fold change >±1.5 for CD4^+^ TEMRAs, Tc17 cells, CD103^+^CLA^−^ cells within the CD4^+^ T cell population, CD4^+^ TEM cells, CD4^+^ TCM cells, Th17 cells, and Tc22 cells (Figure 2B).

Following identification of differences in T cell subset composition among the patient subgroups, T cell subset frequencies were subsequently compared using Kruskal–Wallis test, followed by Dunn's post hoc analysis to assess pairwise differences. The statistical results, illustrated in Figure 2C, unveiled significant differences in T cell subset frequencies among the patient subgroups. Notably, Th17 cells [H (2) = 8.47, *P* = 0.0145] were decreased in subclinical psoriatic arthritis compared to psoriatic arthritis. By contrast, Tc17 cells [H (2) = 8.43, *P* = 0.0148] were increased in subclinical psoriatic arthritis compared with psoriasis and psoriatic arthritis, as were CD4^+^ TEM cells [H (2) = 10.09, *P* = 0.0064].

To assess therapeutic effects on T cell subset frequencies, 114 patients underwent immunophenotyping at therapy initiation and after 2, 4, and 16 weeks (Supplementary Figure 6). Significant differences in T cell subset frequencies were observed depending on the therapy and timepoint. Within patients treated with targeted synthetic DMARDs, Th17 cells differed significantly between therapy initiation (week 0) and week 16 as well as weeks 2 and 16, but not between weeks 4 and 16. In patients treated with csDMARDs, Th22 cells differed between therapy initiation and week 16. For IL‐(12)/23 inhibitors, significant increases were observed in Th22 and T helper granulocyte–macrophage colony‐stimulating factor CD4 T cells, respectively. Additionally, a significant increase in Th22 cell frequencies occurred under IL‐17(R) inhibitors between therapy initiation and week 16. However, no consistent shifts across therapies or persistent changes over time were identified.

### Distinct T cell phenotype and higher frequencies of Tc17 cells in patients with enthesitis

Having found differences in T cell subset frequencies among patients with psoriasis, subclinical psoriatic arthritis, and definitive psoriatic arthritis, we reasoned that entheseal inflammation could also be driven by changes in T cell subset composition during transition. Therefore, T cell phenotyping was performed in patients with subclinical and definitive psoriatic arthritis. Within the subgroup of patients with subclinical psoriatic arthritis, four of eight patients (50.00%) showed imaging‐confirmed entheseal alterations without clinical signs of enthesitis. Among patients with psoriatic arthritis, 41 of 62 patients (66.13%) were diagnosed with enthesitis. In contrast, none of the patients with psoriasis showed clinical signs of enthesitis. Thus, the subgroup with psoriasis was excluded from analysis. The peripheral blood T cell phenotype and frequencies of individual T cell subsets were assessed and compared between patients with and without enthesitis.

**Figure 3:**
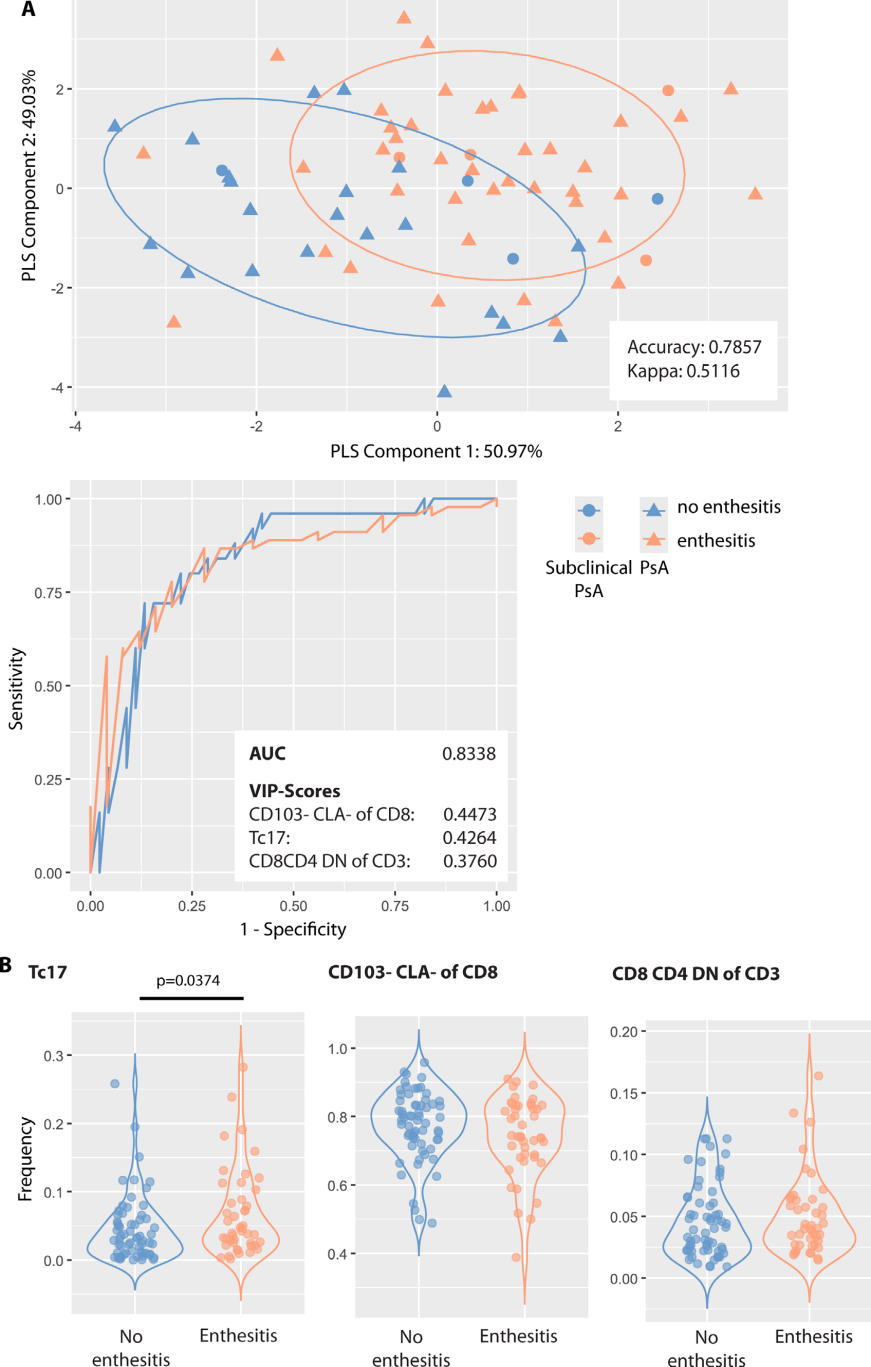
Patients with enthesitis have a distinct T cell phenotype and display higher levels of Tc17 cells. (A) Partial Least Squares (PLS)‐Discriminant Analysis distinguished patients with enthesitis based on their peripheral blood T cell phenotype achieving an accuracy of 0.7859. Patients with enthesitis are depicted in orange, whereas those without enthesitis are shown in blue. Further, patients with subclinical psoriatic arthritis (PsA) are indicated by dots, and those with PsA are represented by triangles. The receiver operating characteristic analysis for predicting enthesitis using T cell subsets yielded an area under the curve (AUC) of 0.8338. Key discriminative T cells identified by the variable importance (VIP) score include CD103^−^cutaneous Ly (CLA)^−^ cells within the CD8^+^ T cell population, Tc17 cells, and CD8^−^CD4^−^ double‐negative (DN) T cells. (B) Violin plots illustrate the distribution of T cell frequencies for the three most influential subsets as identified by the VIP score in both enthesitis and nonenthesitis groups. The Mann–Whitney *U* test disclosed a statistically significant difference for Tc17 frequencies between the two groups (*P* = 0.0374). However, no significant differences were found in frequencies of CD103^−^CLA^−^ cells within the CD8^+^ T cell population and CD8^−^CD4^−^ DN T cells.

PLS‐DA differentiated patient subgroups based on their blood T cell phenotype, achieving an accuracy of 0.7859, as shown in Figure 3A. Receiver operating characteristic (ROC) analysis for predicting enthesitis using T cell subsets yielded an area under the curve (AUC) of 0.8338. The variable importance (VIP) score identified key discriminative T cell subsets including CD103^−^CLA^−^ cells within the CD8^+^ T cell population, Tc17 cells, and CD8^−^CD4^−^ double‐negative (DN) T cells. Further analysis using the Mann–Whitney *U* test compared the frequencies of these top three T cell subsets between patients with and without enthesitis. A higher frequency of Tc17 cells was found in patients with enthesitis compared to patients without enthesitis (*P* = 0.0374; Figure 3B). By contrast, no significant differences were found for the frequencies of CD103^−^CLA^−^ cells within the CD8^+^ T cell population and CD8^−^CD4^−^ DN T cells between patients with and without enthesitis.

### CCR5 ^+^, CCR6 ^+^ (Tc17) cells as key cells differentiate among patients with psoriasis, subclinical psoriatic arthritis, and psoriatic arthritis

**Figure 4:**
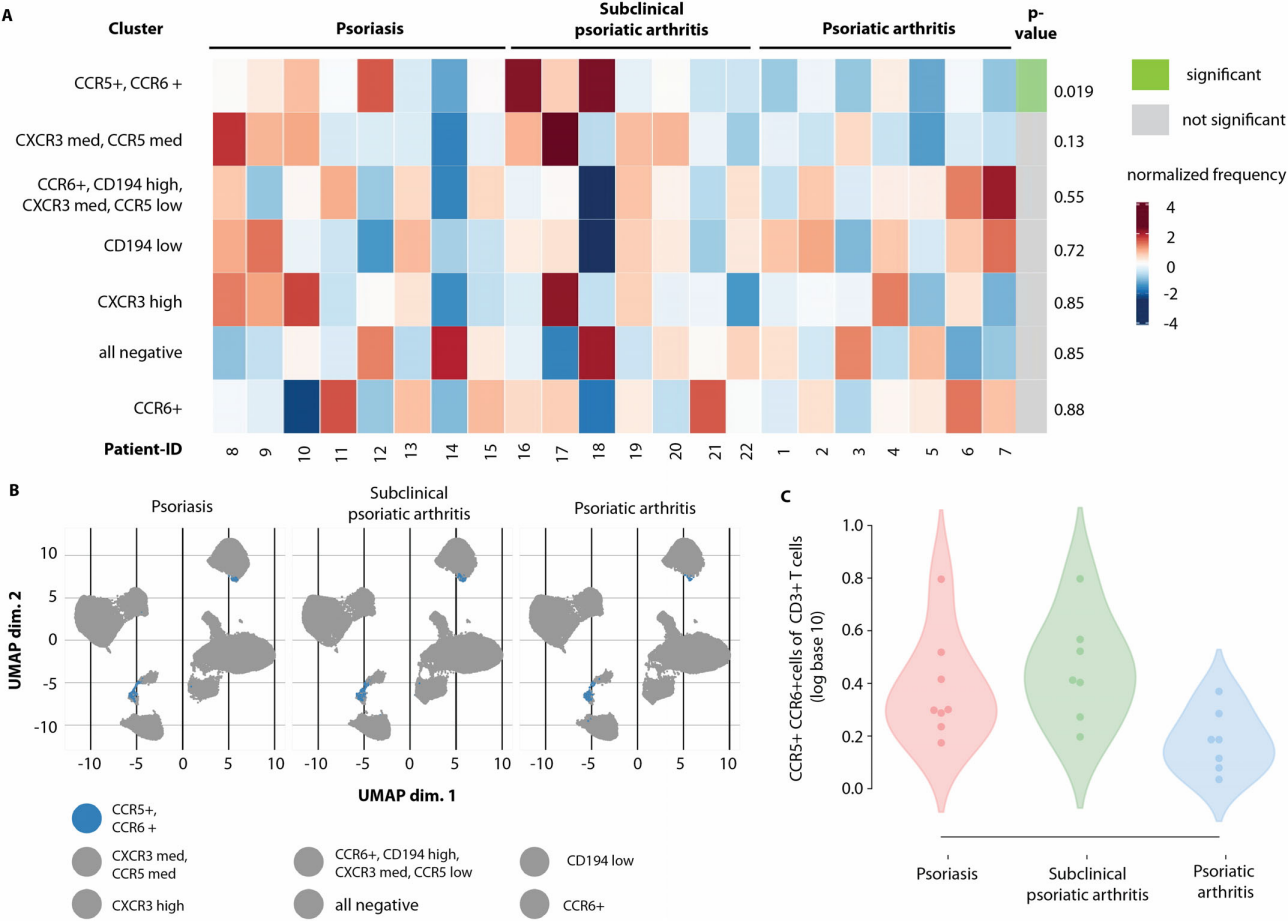
Machine‐learning–based analysis reveals CCR5^+^, CCR6^+^ (Tc17) cells as key to differentiate among patients with psoriasis, subclinical psoriatic arthritis, and psoriatic arthritis. (A) Diffcyt plot illustrating differential abundance of (CCR5^+^, CCR6^+^) Tc17 cells in the prodromal psoriatic arthritis group at false discovery rate >0.05. (B) Significantly altered metacluster highlighted in uniform manifold approximation and projection (UMAP) plot split by condition. (C) Application of gating strategy informed by analysis of metacluster CCR5^+^, CCR6^+^ (Tc17). The plot shows results of reanalysis of patients using this gating strategy. Significance was assessed using Kruskal–Wallis test. dim., dimension; med, medium.

To confirm our findings suggesting a major role for Tc17 cells in driving transition from psoriasis to psoriatic arthritis, we employed nonsubjective ML‐assisted unsupervised clustering as a flow cytometry data readout strategy. The optimal clustering depth identified a total of 15 metaclusters from two panels (Supplementary Figure 7). UMAP parameters were adjusted to optimally display metaclusters, and annotations were performed as described in the Materials and Methods section. The resultant lymphocyte maps of both datasets employed in the study are shown in Supplementary Figure 8A. Further, Supplementary Figure 8B shows the heatmaps of lymphocyte marker expression of all clusters in both datasets as well as their abundance. The estimated spectrum of ML‐detectable cell subsets is summarized in Supplementary Table 3. Dataset 1 included CD4^+^ naïve T cells (TN), TCM CD4^+^, TEM CD4^+^, TN CD8^+^, TEM CD8^+^, and DN cells. In panel 2, DN, Tc17, Tc1, Tc2, Th17, and Th2 cells were detected. Subsequently, the abundance of metaclusters in the three groups was assessed. In Supplementary Figure 8C, plots display abundance of metaclusters split by groups. We employed differential abundance analysis of metaclusters as described in the Materials and Methods. Figure 4A shows that CCR5^+^, CCR6^+^ (Tc17) cells were significantly increased in subclinical psoriatic arthritis compared to the two other subgroups (at false discovery rate > 0.05). This was not the case for all other metaclusters. Figure 4B displays UMAP plots split by groups with the significant population of CCR5^+^, CCR6^+^ (Tc17) cells highlighted. Backgating of metacluster CCR5^+^, CCR6^+^ (Tc17) confirmed that patients with subclinical psoriatic arthritis showed increased frequencies of Tc17 cells compared to patients with psoriasis at risk of transition and psoriatic arthritis (Figure 4C).

### Random Forest analysis yields high accuracy and robustness in classification of patients

**Figure 5:**
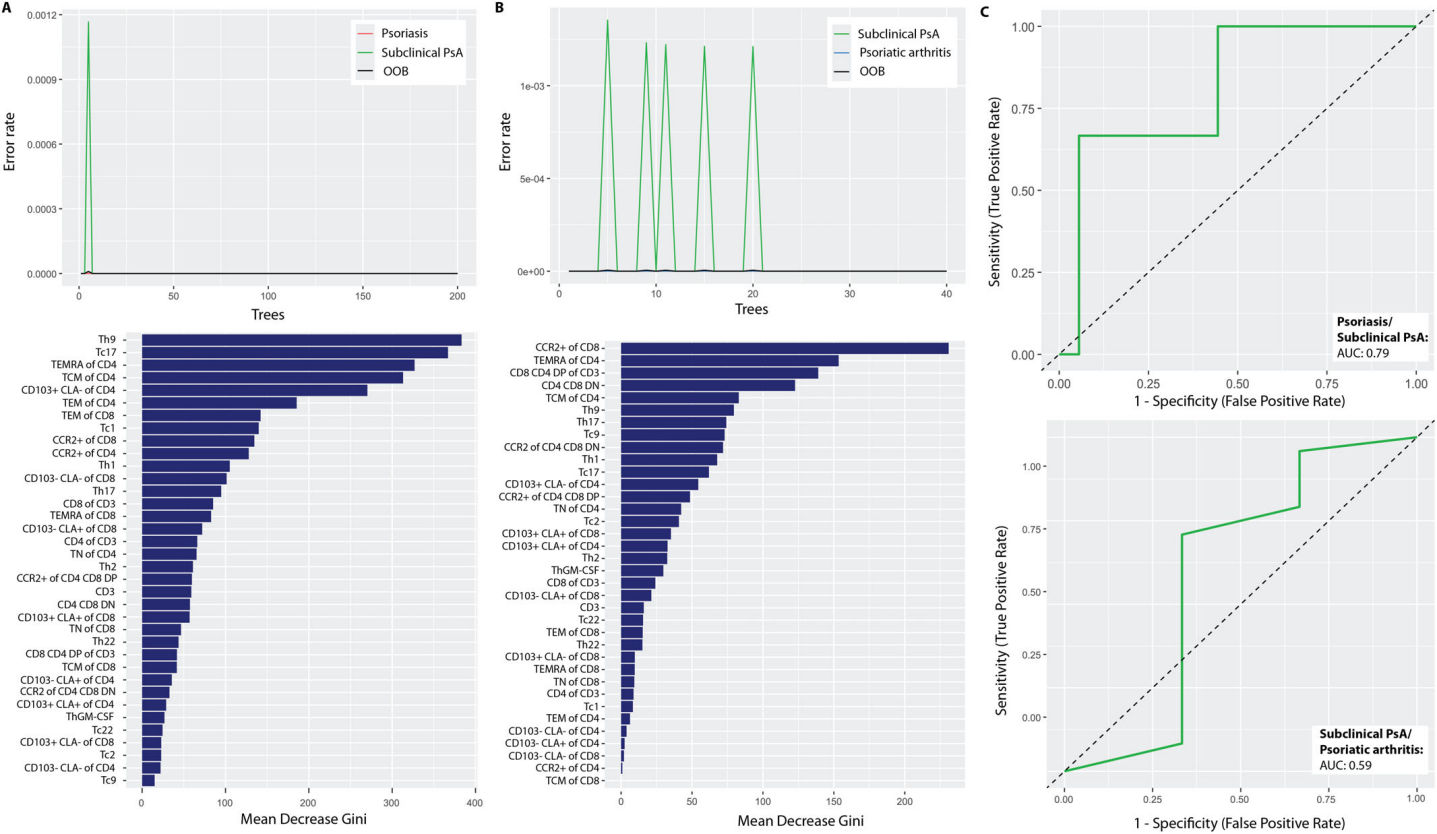
Random Forest analyses reveal a high accuracy and robustness in classification of patients. Results of Random Forest analyses for classification of patients into having (A) psoriasis and subclinical psoriatic arthritis (PsA) or (B) subclinical PsA and PsA are shown as error rate plots visualizing the error rate of the Random Forest model as the number of trees increased with red representing psoriasis, green subclinical PsA, and blue PsA. The out‐of‐bag error (OOB) is depicted in black. The variable importance plots depict the importance of individual T cell subsets based on the Mean Decrease in Gini, helping to identify which features were most influential in both models. (C) Results of the receiver operating characteristic analysis of the two Random Forest analyses in the test dataset are depicted. Area under the circle (AUC) values are shown in the legends beside the respective model. CLA, cutaneous Ly; DN, double negative; DP, double positive; TCM, central memory T; TEM, effector memory T; TEMRA, TEM cells re‐expressing CD45RA; ThGM‐CSF, T helper granulocyte–macrophage colony‐stimulating factor; TN, naïve T cells.

To further assess T cell subset–based stratification of psoriatic disease, we performed Random Forest analyses aimed at distinguishing between psoriasis and subclinical psoriatic arthritis, as well as between subclinical and established psoriatic arthritis. The models incorporated T cell subset frequencies, revealing high accuracy and robustness in classification performance. For comparison of psoriasis versus subclinical psoriatic arthritis, the best‐performing model (mtry 6, ntree 200, nodesize 4) achieved an accuracy of 1.000 in the training data, with a balanced accuracy of 69.44% on the test set. VIP analysis indicated that Th9, Tc17, and TEMRAs within the CD4^+^ T cell population were critical for patient classification, underscoring their role during transition. In distinguishing subclinical psoriatic arthritis from established psoriatic arthritis, the optimal model (mtry 7, ntree 40, nodesize 3) also reached 100% accuracy in the training set and 81.48% on the test data. Here, CCR2^+^ CD8^+^ T cells, TEMRAs within the CD4^+^ T cell population, and CD4^+^CD8^+^ double‐positive CD3^+^ T cells emerged as top contributors, reflecting progressive immune activation and tissue involvement. Figure 5A and B presents error rate and VIP plots, revealing T cell subsets driving the classification.

Figure 5C illustrates the ROC analysis for the test dataset based on both Random Forest analyses. For the classification of patients into psoriasis or subclinical psoriatic arthritis, the AUC was 0.79. For distinguishing between patients with subclinical psoriatic arthritis and psoriatic arthritis, the AUC was 0.59 in the test dataset. These findings suggest an adequate discriminative ability for identifying patients with subclinical psoriatic arthritis within the group of patients with psoriasis, at the same time highlighting the challenge in differentiating between patients with subclinical and definitive psoriatic arthritis.

## DISCUSSION

Strong MHC class I association, increased frequencies of circulating and tissue‐infiltrating cytokine‐producing T cell subsets including Th17 cells, and the efficacy of biologic therapies targeting TNFα and the IL‐23/IL‐17 axis suggest a role of T cells as key drivers of immunopathology in psoriasis and psoriatic arthritis.^8^, ^9^, ^10^, ^11^, ^12^, ^13^, ^14^, ^15^, ^16^, ^29^, ^30^ However, knowledge is lacking with respect to changes of the T cell compartment driving transition from psoriasis to psoriatic arthritis and providing a link between cutaneous and joint inflammation, which affects about 30% of the patients with psoriatic disease.^17^, ^31^ Apart from a study by Gao et al^32^ reporting a significantly higher Th1‐to‐Th2 ratio in patients with psoriatic arthritis compared to those without, studies on changes within the peripheral blood T cell compartment potentially driving transition from psoriasis to psoriatic arthritis are lacking. Therefore, we determined changes within the peripheral blood T cell compartment using deep T cell phenotyping in the transition phase in this study.

Consistent with the role of T cells in psoriatic disease,^8^, ^9^, ^10^, ^11^, ^12^, ^13^, ^14^, ^15^, ^16^, ^29^, ^30^ we found overlapping T cell endotypes among patient subgroups with psoriasis, subclinical psoriatic arthritis, and psoriatic arthritis, albeit with the transition group (ie, patients with subclinical psoriatic arthritis exhibiting the most notable divergence from the two other subgroups). Earlier studies reported increased frequencies of circulating cytokine‐producing Th1, Th17, and Th22 helper cell subsets and enrichment of Th1, Th9, Th17, and Th22 cells and their Tc1, Tc17, and Tc22 cytotoxic T cell counterparts in psoriatic lesions. Moreover, IL‐17‐producing CD8^+^ T cells are present in synovial fluid of affected joints in psoriatic arthritis.^10^, ^11^, ^12^, ^13^, ^14^, ^15^, ^16^, ^29^, ^30^, ^33^, ^34^ Notably, Th17 cells differ from their Tc17 cell counterparts regarding chemokine receptor expression and, thus, responsiveness to chemokine attractants. In contrast to Tc17 cells, Th17 cells express CCR4, a receptor for various chemokines including Cystein‐Cystein (CC)‐chemokine ligand CCL17 expressed in cutaneous vessels and epidermis.^35^ Whereas the above mentioned study by Gao et al included patients with psoriasis and psoriatic arthritis,^32^ our study comprised three different subgroups including patients with psoriasis, subclinical psoriatic arthritis according to EULAR definition, and definitive psoriatic arthritis according to CASPAR criteria, thereby representing the whole spectrum ranging from pre‐ to posttransitional psoriatic disease. In the present study, using deep immunophenotyping, we found significant differences in circulating CD4^+^ TEM, Th17, and Tc17 cell frequencies among patients with psoriasis, subclinical psoriatic arthritis, and psoriatic arthritis suggestive of IL‐17‐producing T cells as major drivers of transition from psoriasis to psoriatic arthritis. In particular, a decrease in Tc17 cell frequency in patients with psoriatic arthritis in comparison to patients with subclinical psoriatic arthritis suggests cell migration into joints during transition to arthritis. Notably, migration of TEM cells toward sites of inflammation during active disease resulting in decreased frequency of peripheral blood TEM cells has been shown in various chronic inflammatory diseases.^36^, ^37^ CD4^+^ and CD8^+^ TEM cells migrate from peripheral blood to peripheral tissues, where they either remain permanently as long‐lived tissue‐resident memory (TRM) cells or from where they recirculate at a later time point.^38^, ^39^ Circulating Tc17 cells infiltrate sites of inflammation and are enriched in psoriatic skin lesions and in synovial fluid in psoriatic arthritis joints.^20^, ^33^, ^40^ Moreover, Tc17 cells can convert to long‐lived skin‐resident TRM cells expressing the skin‐homing sialyl‐LewisX glycoprotein CLA and αE integrin‐subunit (CD103).^15^, ^16^ In contrast to Th17 cells, skin‐resident Tc17 cells lack therapeutic responsiveness to narrowband‐UV‐B and TNFα and IL‐23/IL‐17 axis–inhibiting biologics. They are retained in resolved lesions, thereby conferring a pathologic site‐specific disease memory potentially driving recurrent psoriasis and facilitating transition from psoriasis to psoriatic arthritis.^20^ In addition, recirculation of originally skin‐resident CD103^+^ TRM cells may perpetuate inflammation at distant sites.^41^ In the present study, however, we found no significant differences in circulating CLA^+^CD103^+^ TRM cell frequencies among patients with psoriasis, subclinical psoriatic arthritis, and psoriatic arthritis. This does not exclude a role for TRM cell recirculation in transition and disease progression. Similar to the study by Klicznik et al,^41^ frequencies of circulating TRM cells were low in all three patient groups of our study and did not allow a conclusion with regard to their potential to reenter the circulation and migrate to other sites.

The potential predominance of IL‐17‐producing T cells and in particular Tc17 cells in driving transition from psoriasis to psoriatic arthritis is further supported by our finding of higher Tc17 frequencies in those patients displaying imaging‐confirmed entheseal alterations without clinical signs of enthesitis in subclinical psoriatic arthritis and enthesitis in definitive psoriatic arthritis. Entheseal inflammation commonly affects patients with subclinical and definitive psoriatic arthritis and is regarded as a prognostic factor of imminent transition from subclinical to clinical manifest psoriatic arthritis.^23^ Comparable with other studies,^42^, ^43^ one‐third of the patients with subclinical psoriatic arthritis and roughly half of the patients with definitive psoriatic arthritis were affected by enthesitis in our study cohort. A spondylarthritis animal model suggests a role of the IL‐23/IL‐17 axis for synovioentheseal inflammation.^44^ Entheseal‐resident CD4^+^ and CD8^+^ T cells have been reported to produce TNF, but IL‐17A production was solely confined to CD4^+^ T cells.^45^ However, Tc17 cells display high plasticity and can switch to Tc1 cells upon recruitment from peripheral blood into tissues.^46^

This study is the first to investigate the immunologic characteristics of patients representing a spectrum of psoriatic disease in transition from psoriasis to psoriatic arthritis in a cross‐sectional study. A major strength of the study lies in the precise clinical characterization of the patients.^8^, ^47^ Nevertheless, misclassification of individual patients cannot be ruled out because psoriatic disease displays a disease spectrum for which there are currently no clear distinctions between the individual disease phases. Patients presenting with musculoskeletal symptoms and imaging‐confirmed enthesopathy lacking overt clinical swelling can be at risk of misclassification because they are in a diagnostic gray area between subclinical and definite psoriatic arthritis. The use of a structured ultrasound scoring system for entheseal assessment could improve diagnosis of enthesitis.^48^ There is also a need for further clinical and diagnostic tools as well as biomarkers to accurately stratify patients in the disease stages. Our findings suggest that Th17, Tc17, and CD4^+^ TEM cells should be investigated for their applicability in diagnostic algorithms for early diagnosis of subclinical psoriatic arthritis. Limitations of our study include its cross‐sectional study approach and, thus, lack of individual long‐term longitudinal follow‐up of changes in T cell composition during transition, potentially biasing the results A further limitation of this study includes the differences in size among the three patient subgroups. However, to compensate for these shortcomings and further validate our findings, we performed ML‐assisted readout of flow cytometry data, thereby confirming the main finding of an increased abundance of Tc17 cells in subclinical psoriatic arthritis. As previously shown, ML‐assisted flow cytometry readout adds to reproducibility of flow cytometry results.^49^ To confirm the results of this study, validation in a larger cohort is required, which may be achieved by a multicenter study design, as well as a longitudinal design, further supported by analysis of tissue samples.

In summary, our findings suggest a role for CD4^+^ TEM, Th17, and especially Tc17 cells potentially migrating to joints and entheses during transition. Accurately identifying patients at risk of transition from psoriasis to psoriatic arthritis, and understanding T cell compartment changes that drive this process, is crucial to determine the window of opportunity for early and effective therapeutic interventions aimed at preventing disease progression and improving patient outcomes.^8^, ^21^ However, lack of therapeutic responsiveness of Tc17 cells^20^, ^33^, ^40^ may favor recurrent inflammation and transition from psoriasis to psoriatic arthritis. Potentially, CCR6 expressed by Th17 and Tc17 cells could be an interesting target to reduce migration of these cells to inflamed tissues in psoriasis and psoriatic arthritis. We conclude that future therapeutic approaches to prevent transition from psoriasis to psoriatic arthritis and to provide more effective therapies or even a cure for psoriatic disease must specifically target the IL‐23/IL‐17 axis and particularly focus on impacting Tc17 cells.

## AUTHOR CONTRIBUTIONS

All authors contributed to at least one of the following manuscript preparation roles: conceptualization AND/OR methodology, software, investigation, formal analysis, data curation, visualization, and validation AND drafting or reviewing/editing the final draft. As corresponding author, Dr Graßhoff confirms that all authors have provided the final approval of the version to be published and takes responsibility for the affirmations regarding article submission (eg, not under consideration by another journal), the integrity of the data presented, and the statements regarding compliance with institutional review board/Declaration of Helsinki requirements.

## Supporting information


**Disclosure form**.

**Appendix S1:** Supplementary Information
