# Supplementary material for: Transition From Psoriasis to Psoriatic Arthritis is Characterized by Distinct Alterations in Peripheral Blood Tc17, Th17, and CD4 + Effector Memory Cells
Source: Arthritis Rheumatol. 2025 Dec 28;78(2):332–43. doi: 10.1002/art.43396 (PMC12936891; doi:10.1002/art.43396)
Supplement: Supplementary file 2 — Appendix S1: Supplementary Information [file ART-78-332-s002.docx]

**Supplementary Material**

**Ethical approval and study design**

Ethical approval was obtained from the local ethics committee (AZ20-170A). All patients gave informed consent to participate in the study. Consecutive patients with psoriasis, in the transition phase and with psoriatic arthritis were included between 19^th^ June 2020 and 18^th^ January 2023 and were followed up at the comprehensive center for inflammation medicine and department of rheumatology and clinical immunology at the UKSH, campus Lübeck. The diagnosis of *psoriasis* and nail involvement was made clinically by a dermatologist. Inclusion criteria for psoriasis patients were i) active disease with an indication for initiation of systemic therapy according to the 2021 S3 guideline for the treatment of psoriasis ^1^ and ii) *at* *least one* *risk factor for transition to psoriatic arthritis* ^2,3^. According to Zabotti et al., risk factors constitute nail involvement, obesity, severe psoriasis, and another affected individual among first-degree relatives with psoriatic arthritis ^4^. The subgroup of psoriasis patients with at least one risk factor for transition to psoriatic arthritis is termed ‘psoriasis’ in the following. *Subclinical psoriatic arthritis* was defined as psoriasis with arthralgia and/or imaging evidence of synovial/entheseal inflammation without clinical synovitis as suggested by a systemic literature review of a EULAR taskforce in 2023 ^5^. In this context, pain, joint tenderness, arthralgia, and imaging-determined enthesopathy without clinical signs of enthesitis assessed by a rheumatologist were regarded as predictors of imminent transition to psoriatic arthritis. *Psoriatic arthritis* was diagnosed and classified according to the CASPAR criteria by a rheumatologist ^6^. Inclusion criteria for patients with psoriatic arthritis were fulfillment of CASPAR criteria and active disease with an indication for initiation of systemic therapy according to current EULAR and GRAPPA recommendations for the treatment of psoriatic arthritis ^7,8^. Patients included with psoriasis, subclinical psoriatic arthritis and psoriatic arthritis had either not previously been treated with systemic therapy or had not responded to the previous treatment approach. Individuals who were pregnant or actively lactating were excluded from the study.

**Patient and baseline information**

Demographic parameters, clinical data, and disease activity scores were assessed. Psoriasis Area severity Index (PASI) was used to assess psoriatic disease severity and the Dermatology Life Quality Index (DLQI) for the assessment of impact on quality of life. Itch was reported using a visual analogue scale ranging from 0 to 10 ^9^.

Joint involvement was assessed by evaluating 68 joints for tenderness and 66 joints for swelling according to the Disease Activity in Psoriatic Arthritis score (DAPSA). Arthritis was categorized by the number of swollen joints into monarthritis (n=1), oligoarthritis (n=2-4), and polyarthritis (n≥5) ^10^. Furthermore, all psoriasis patients at risk for transition were screened for psoriatic arthritis using the GErman Psoriasis ARthritis Diagnostic (GEPARD) questionnaire. A score ≥4 on the 13-item GEPARD questionnaire suggests the presence of psoriatic arthritis. A diagnosis of psoriatic arthritis was finally confirmed in one-third of the patients referred to a rheumatologist, with higher diagnostic rates observed in hospital settings (43.7%) compared to private practices (25.8%) ^11,12^. Diagnosis of enthesitis was made clinically assessing entheses according to the Leeds enthesitis index and the Maastricht Ankylosing Spondylitis Enthesitis ^13^. In case of clinically absent signs of enthesal tenderness and swelling, enthesal diagnostics were supported by ultrasound and MR imaging in patients with subclinical psoriatic arthritis. The standard ultrasound evaluation incorporated entheses examinations of the Achilles tendon, the plantar fascia, the inferior and superior poles of the patella, and the patellar ligament insertion at the tibial tuberosity. In addition, complaint-related ultrasound assessments were performed as needed. Ultrasound signs like hypoechogenicity or thickening within 2 mm of the bony cortex, Power Doppler Signal, erosions or enthesophytes were interpreted as signs for active inflammatory enthesitis ^14,15^. In case of imaging-proofed enthesal alterations without reporting corresponding symptoms, the patients were subgrouped as ‚image-determined enthesiopathy without clinical signs of enthesitis’. Axial disease was diagnosed based on X-ray or MR imaging of the spine. Dactylitis was diagnosed clinically and supported by ultrasound. Pain was reported using a visual analogue scale ranging from 0 to 10. Nail psoriasis was defined by distal onycholysis, hyperkeratosis or pitting. Disability due to inflammatory joint disease was assessed by Health assessment questionnaire (HAQ) ^16^. Current uveitis or diagnosis of uveitis in the past, family history and previous therapy were recorded according to the patients' self-report and on the basis of previously chart-recorded findings. Absolute number of lymphocytes was determined by differential blood count.

Details of the diagnostic pathway to classify study participants are shown in **Suppl. Figure 1**.

**Deep T cell phenotyping by multicolor flow cytometry**

Deep T cell phenotyping was performed in all 116 patients. Heparinized blood samples were collected from the patients and subsequently analyzed by multicolor flow cytometry. Red blood cells were lysed using 1 x RBC lysis buffer (#420302, BioLegend, USA) according to manufacturer’s protocol. Cells were washed twice with staining buffer (0.5% Bovine Serum Albumin (BSA) in PBS). Fc-receptors were blocked with TruStainFcX™ (#422302, BioLegend, USA) in 50 μL staining buffer for 5 minutes at RT and dead cells were excluded using Zombie NIR™ Fixable Viability Kit (#423105, BioLegend, USA) according to the manufacturer`s protocol. Cells were stained for 15 minutes at room temperature using the fluorophore labelled antibodies listed in **Suppl. Table 1** (2 μL each per sample). After staining, the cells were washed and resuspended in 300 μL 0.5% BSA in PBS. Subsequently, the samples were acquired using CytoFLEX S spectral analyser flow cytometer (Beckman Coulter, USA) and CytExpert software (Beckman Coulter, USA).

Data analysis was performed using FlowJo v10 (BD Biosciences, USA). Doublets were excluded by FSC-H and FSC-A. Lymphocytes were identified by size (FSC-A) and granularity (SSC-A). Live T cells were gated as Zombie-CD3+ and further divided into T cell subsets according to the expression of CD4 and CD8. T cell subsets were characterized by deep T cell phenotyping including chemokine receptor expression using gating strategy according to Mousset et al. ^17^ and Staser et al. ^18^ (**Suppl. Figure 2**). Th17 cells were identified within the CD4+ CD8- T cell population as CCR6+ CCR4+ CCR10- cells, whereas Tc17 cells were gated from the CD4- CD8+ population as CCR6+ CCR5+ CCR10- cells. Both Th17 and Tc17 cells express the CC motif chemokine receptor (CCR)6 playing a key role in the migration of both cell types into inflamed tissue ^19,20^. Notably, Th17 and Tc17 cells differ regarding CC motif chemokine receptor (CCR)4 expression with the former expressing CCR4 and the latter not ^17^.

Circulating tissue-resident memory T cells (TRM) were identified based on surface expression of cutaneous lymphocyte antigen (CLA) and integrin α-E (CD103). CD103 pairing with integrin β7 was assessed due to its role in binding E-cadherin and promoting tissue migration. CLA, as part of PSGL-1, was evaluated for its interaction with P- and E-selectins, relevant for TRM skin homing ^21^. Gating strategies are shown in **Suppl. Figure 3**.

To further investigate the impact of therapeutics on the frequency of T cell subsets, 114 patients with psoriasis, subclinical psoriatic arthritis and psoriatic arthritis were followed up via immunophenotyping at timepoint of therapy initiation as well as two, four and sixteen weeks after induction of systemic therapy. Patients were grouped according to initiation of csDMARDs, tsDMARDs, TNF inhibitors, IL-17(R) inhibitors or IL-(12)/23 inhibitors. The frequency of T cells was compared on the one hand between different timepoints and on the other hand between the respective therapies at each timepoint using Two-Way ANOVA with Tukey’s post hoc test.

**Machine-learning (ML-) based flow cytometry data processing**

To perform a machine-learning based flow cytometry data processing, all events from a subcohort matched by age, sex and BMI (8 patients with psoriasis at risk for transition, 7 subclinical psoriatic arthritis patients, and 7 psoriatic arthritis patients) were combined into one flowset, gated on live lymphocytes using R and meticulously preprocessed (described in the Methods section). Overall, 745,133 events were compiled in the dataset, out of which are 300,000 CD45+CD3+Zombie negative.

The analytical procedures were conducted using the R statistical environment (v4.2). Flow cytometry data were processed using the “FlowCore” (authors: B Ellis, P Haaland, F Hahne, N Le Meur, N Gopalakrishnan, J Spidlen, M Jiang, G Finak, S Granjeaud) and “FlowVS” package (author: A Azad). Data analysis was performed using “CATALYST” (authors: HL Crowell, VRT Zanotelli, S Chevrier, MD Robinson, B Bodenmiller) and “SingleCellExperiment” package (A Lun, D Risso, K Korthauer, K Rue-Albrecht, L Zappia). Data visualization was achieved with “ggplot2” and “ggcyto” (author: M Jiang). Differential abundance analysis was carried out using the “diffcyt” package (author: LM Weber).

We utilized flow cytometry standard (FCS) files that were preprocessed by compensating for spectral overlap and gating for singlets and live cells. We then applied the quality control process implemented with “FlowAI” (authors: G Monaco, H Chen). Subsequently, the data were transformed and scaled using a custom workflow that incorporated FlowVS. Cofactors for arcsin transformation were determined by first performing estParamFlowVS followed by manual inspection and systematical testing of cofactors between 150 and 300,000. The cofactor best capturing known biological peak numbers of a given marker was selected. Then, we performed normalization to mitigate batch effects through an algorithm based on Gaussian normalization (gaussNorm is available in the “flowStats package” (authors: F Hahne, N Gopalakrishnan, A Hadj Khodabakhshi, CJ Wong, K Lee). We then employed the self-organizing map algorithm FlowSOM (authors: S Gassen, A Couckuyt, K Quintelier, A Emmaneel, B Callebaut, Y Saeys) to perform unsupervised clustering of cells based on the expression states of selected markers. In dataset 1 we included CD3, CD4, CD8, CCR7, and CD45RA, and in dataset 2, we focused on CD3, CD4, CD8, CXCR3, CCR5, CCR6 and CCR4. All these markers were classified as state markers, as the cells were pre-gated, excluding type markers. We tested optimal clustering depth by systematically applying ranges of maximum cluster numbers (k) from 10 to 30 as well as grid from 3x3 to 20x20 and analyzing the delta-area plot as well as the multi-dimensional scaling (MDS) plots and arrived at a k of 20 on a 10x10 SOM grid (**Suppl. Fig. 7**). Next, we modified metacluster numbers to generate a clustering balancing fine-granularity while, at the same time, avoiding generation of clusters containing predominantly cells from only one patient.

In this study, we conducted an analysis based on gated lymphocytes with CD3 in the clustering analysis. Cell populations were annotated based on their frequencies and expression profiles according to Mousset et al. ^17^ and Staser et al. ^18^. The assigned long and short names of populations are outlined in **Suppl. Table 3**. Uniform Manifold Approximation and Projection (UMAP) plots were generated using type markers, with a limit of 20,000 cells per sample. We conducted a differential abundance analysis to compare cluster numbers using the diffcyt package. In the final step, we exported the clusters as well as the processed data from R as FCS files and reanalyzed them in comparison with the original data using the flow cytometry software Kaluza (v2.2.1, Beckman Coulter).

**Statistical analysis**

Statistical analysis was performed using the software R version 4.3.3 und GraphPad Prism version 10.0 (GraphPad Software, Inc., USA). Descriptive analysis was used to summarize the demographic and clinical data of the respective patient subgroups. Data are presented as mean (M) ± standard deviation (SD) or frequencies as appropriate. Fisher’s exact test was used to compare the frequency of dichotomous variables with n ≤ 5 within one subgroup. To compare frequencies of dichotomous variables with n>5 in all subgroups, Chi-square test was performed. Continuous variables were tested for normality distribution using Shapiro-Wilk test. To test for differences between two test groups, we used two-sided, unpaired Student’s *t* test or Mann-Whitney *U* test, respectively. Volcano plots were generated using the R package “ggplot2”. To compare three or more test groups, a Kruskal-Wallis test with Dunn’s post hoc test was performed.

Linear regression analysis was used to identify the influence of age and BMI on frequencies of T cell subsets. Data are presented as forest plots displaying standardized estimates and 95% confidence interval (**Suppl. Fig. 4**). To perform linear and logistic regression as well as Partial Least Square - Discriminant Analysis (PLS-DA) data were normalized using the package “bestNormalize” (author: RA Peterson) in R. Afterwards, normality distribution was tested by applying Shapiro-Wilk test and determining skewness and curtosis. Using PLS-DA, the T cell phenotype of patients with psoriasis, subclinical psoriatic arthritis and psoriatic arthritis as well as patients with and without enthesitis was analyzed. To ensure the robustness of the analysis, a 10-fold cross-validation was applied. The PLS-DA was performed using the package “pls” (author: KH Liland, BH Mevik, R Wehrens, P Hiemstra) identifying two components. Using the package “caret” (authors: M Kuhn, J Wing, S Weston, A Williams, C Keefer, A Engelhardt, T Cooper, Z Mayer, B Kenkel, M Benesty, R Lescarbeau, A Ziem, L Scrucca, Y Tang, C Candan, T Hunt) a confusion matrix was provided calculating the accuracy. The package “ggplot2” (authors: H Wickham, W Chang, L Henry, TL Pedersen, K Takahashi, C Wilke, K Woo, H Yutani, D Dunnington, T Brand) was used to visualize these components, with confidence ellipses set at 80% representing different groups and illustrating the separation of the data. VIP (Variable Importance in Projection) scores were calculated using a custom function to determine the significance of each feature in the model. Further analysis included ROC curve evaluation to compare entheseal vs. non-entheseal manifestations. Probability predictions for each class were generated using the predict function from the caret package, and ROC curves were plotted with the pROC package (authors: X Robin, N Turck, A Hainard, N Tiberti, F Lisacek, JC Sanchez, M Müller, S Siegert, Z Billings). The Area Under the Curve (AUC) was computed.

To gain insights into the T cell network, Spearman correlations between T cell subsets were computed using the rcorr function. The results yielded correlation coefficients and p-values, which were examined for significance. To filter relevant correlations, thresholds were set with a Spearman’s r correlation coefficient > 0.7. These data were then extracted as edges. Using the filtered edges, a network was constructed for the T cell subsets for each patient group, namely psoriasis, subclinical psoriatic arthritis and psoriatic arthritis with the “igraph” package (authors: G Csárdi, T Nepusz, V Traag, S Horvát, F Zanini, D Noom, K Müller M Salmon, M Antonov). The network was visualized with edge widths scaled by the -log10 of adjusted p-values according to the Fruchterman-Reingold-Layout algorithm. P-values were adjusted by using False Discovery Rate according to Benjamini-Hochberg.

To perform the Random Forest analyses for both the comparison of psoriasis vs. prodomal psoriatic arthritis and subclinical psoriatic arthritis vs. psoriatic arthritis, the dataset was prepared by imputing missing values with k-nearest neighbours using the package “VIM” (M Templ, A Kowarik, A Alfons, G Cillia, B Prantner, W Rannetbauer). The data was then split into training and test datasets using the package “caret” (60:40), with the training set being balanced using the SMOTE technique from the package “DMwR2” (author: L Torgo). A grid search over the following ranges was conducted (mtry 6-10, ntree 40-300, node size 3-7) combined with 5-fold cross-validation from the “caret” package to optimize the hyperparameters of the Random Forest model using the package “randomForest” (Fortran original by L Breiman and A Cutler, R port by A Liaw and M Wiener). After identifying the best hyperparameters, the final Random Forest model was trained and evaluated, and its performance was visualized using the package “ggplot2” with error rate and variable importance plots. The model's accuracy was finally assessed with a confusion matrix using the package “caret”. Subsequently, ROC analyses were performed by using the “pROC” package for the test datasets. The Area Under the Curve (AUC) values were calculated and the ROC curves were plotted using the package “ggplot2”.


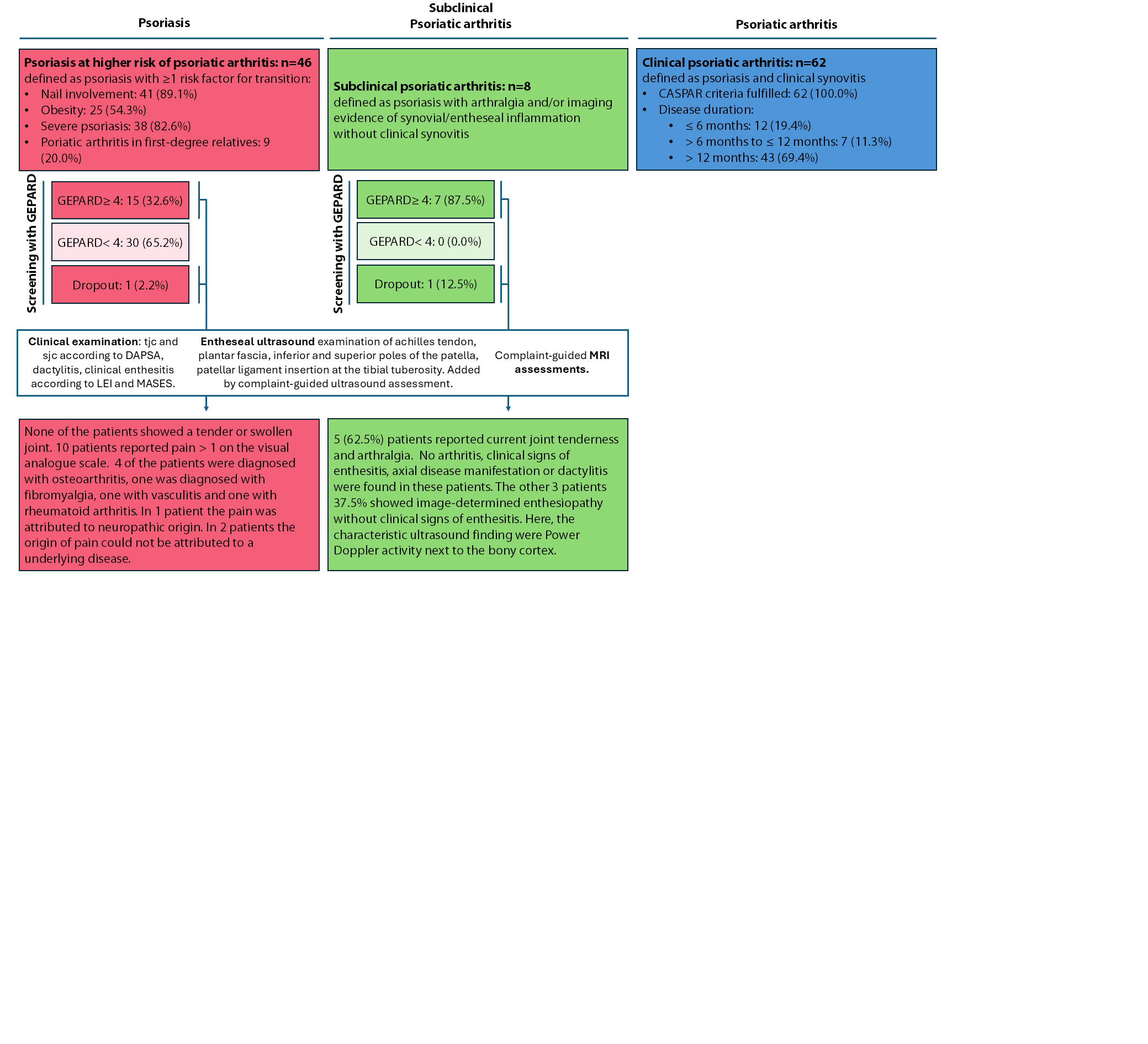


**Suppl. Figure 1**. Classification of study participants. *Abbreviations: CASPAR - ClASsification Criteria for Psoriatic Arthritis, DAPSA - Disease Activity index for PSoriatic Arthritis, GEPARD - GErman Psoriasis ARthritis Diagnostic, LEI - Leeds enthesitis index, MASES - Maastricht Ankylosing Spondylitis Enthesitis, MRI - Magnetic Resonance Imaging.*

| **Marker** | **Fluorophore** | **Clone** | **Supplier** | **Cat.No** |
| --- | --- | --- | --- | --- |
| CD3 | AF 700 | UCHT1 | BioLegend, USA | #300424 |
| CD4 | PerCP/Cy5.5 | RPA-T4 | BioLegend, USA | #300530 |
| CD8a | BV510 | RPA-T8 | BioLegend, USA | #301048 |
| CD45RA | AF 488 | HI100 | BioLegend, USA | #304114 |
| CCR5 | AF 488 | J418F1 | BioLegend, USA | #359104 |
| CCR7 | BV421 | G043H7 | BioLegend, USA | #353208 |
| CCR6 | BV421 | G034E3 | BioLegend, USA | #353408 |
| CCR4 | PE/Cy7 | L291H4 | BioLegend, USA | #359410 |
| CXCR3 | BV650 | G025H7 | BioLegend, USA | #353730 |
| CCR10 | PE | 6588-5 | BioLegend, USA | #341504 |
| CCR2 | BV421 | K036C2 | BioLegend, USA | #357210 |
| CD103 | PE | Ber-ACT8 | BioLegend, USA | #350206 |
| CLA | AF 647 | HECA-452 | BioLegend, USA | #321310 |

**Suppl. Table 1. Markers for identification of T helper (Th) and T cytotoxic (Tc) cells, CD4+ and CD8+ T cell subsets by multicolor flow cytometry.** Fluorophore, Clone, Supplier and Cat. No. are depicted.


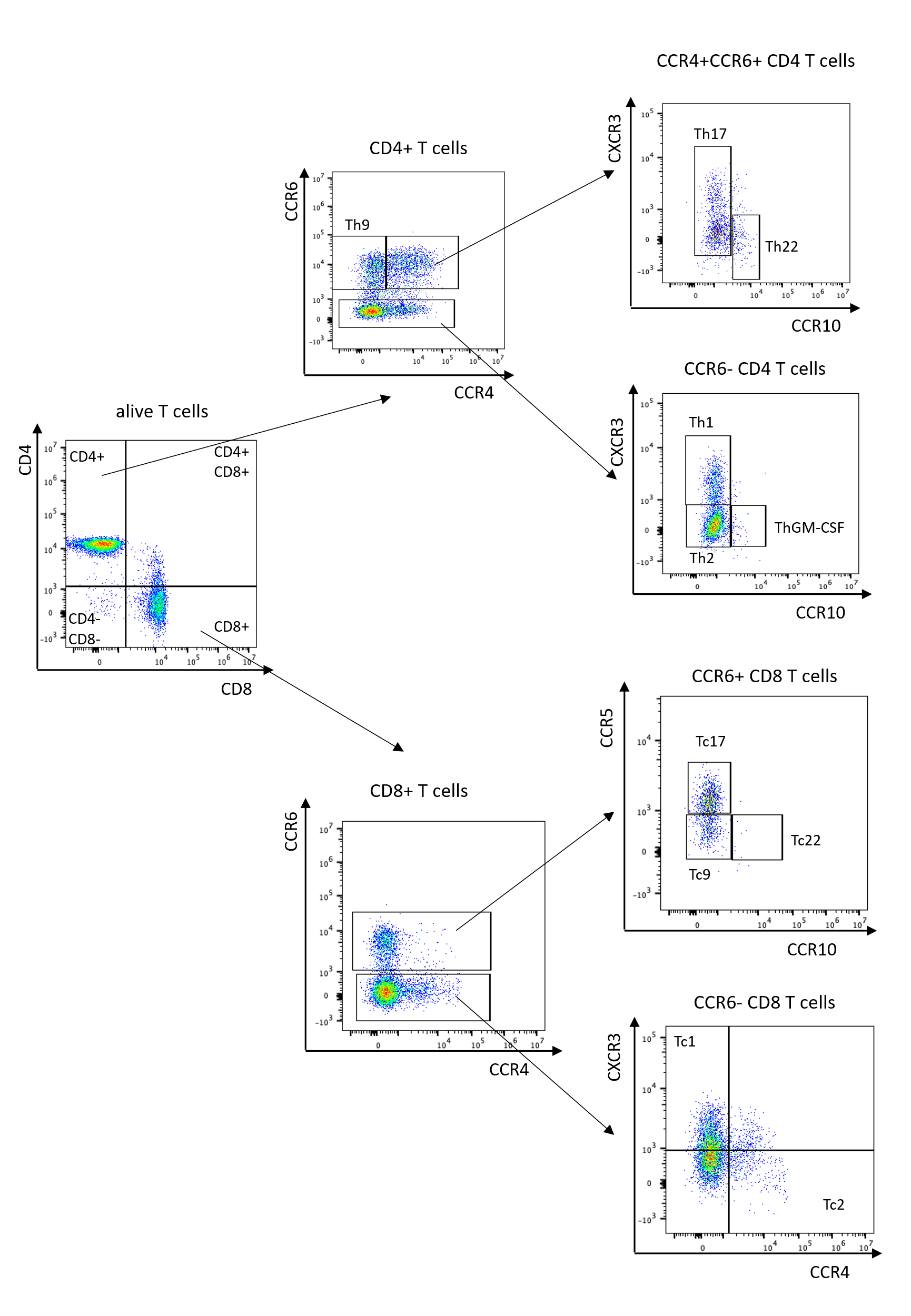


**Suppl. Fig. 2.** **Identification of T helper (Th) and T cytotoxic (Tc) subsets by multicolor flow cytometry**. T cells were separated by CD4+ and CD8+ status and subject to deeper phenotype analysis by chemokine receptor expression according to Mousset et al. ^17^ and Staser et al. ^18^. CD4+CD8- T cells were gated into CCR6-, CCR6+CCR4- (Th9) and CCR6+CCR4+ cell populations. The CCR6- population was further gated into CXCR3+CCR10- (Th1), CXCR3-CCR10- (Th2) and CXCR3-CCR10+ (ThGM-CSF) cell subsets. The CCR6+CCR4+ population was gated into CCR10- (Th17) and CCR10+ (Th22) cells. CD4-CD8+ T cells were gated into CCR6+ and CCR6- cell populations. CCR6+ cells were gated into CCR5+CCR10- (Tc17), CCR5-CCR10- (Tc9) and CCD5-CCR10+ (Tc22) cell subsets. CCR6- cells were gated into CXCR3+CCR4- (Tc1) and CXCR3-CCR4+ (Tc2) cell subsets. Notably, Th17 and Tc17 cells differ regarding CCR4 expression with the former expressing CCR4 and the latter not ^17^. Therefore, gating strategies based on chemokine receptor expression differ between Th and Tc subsets.


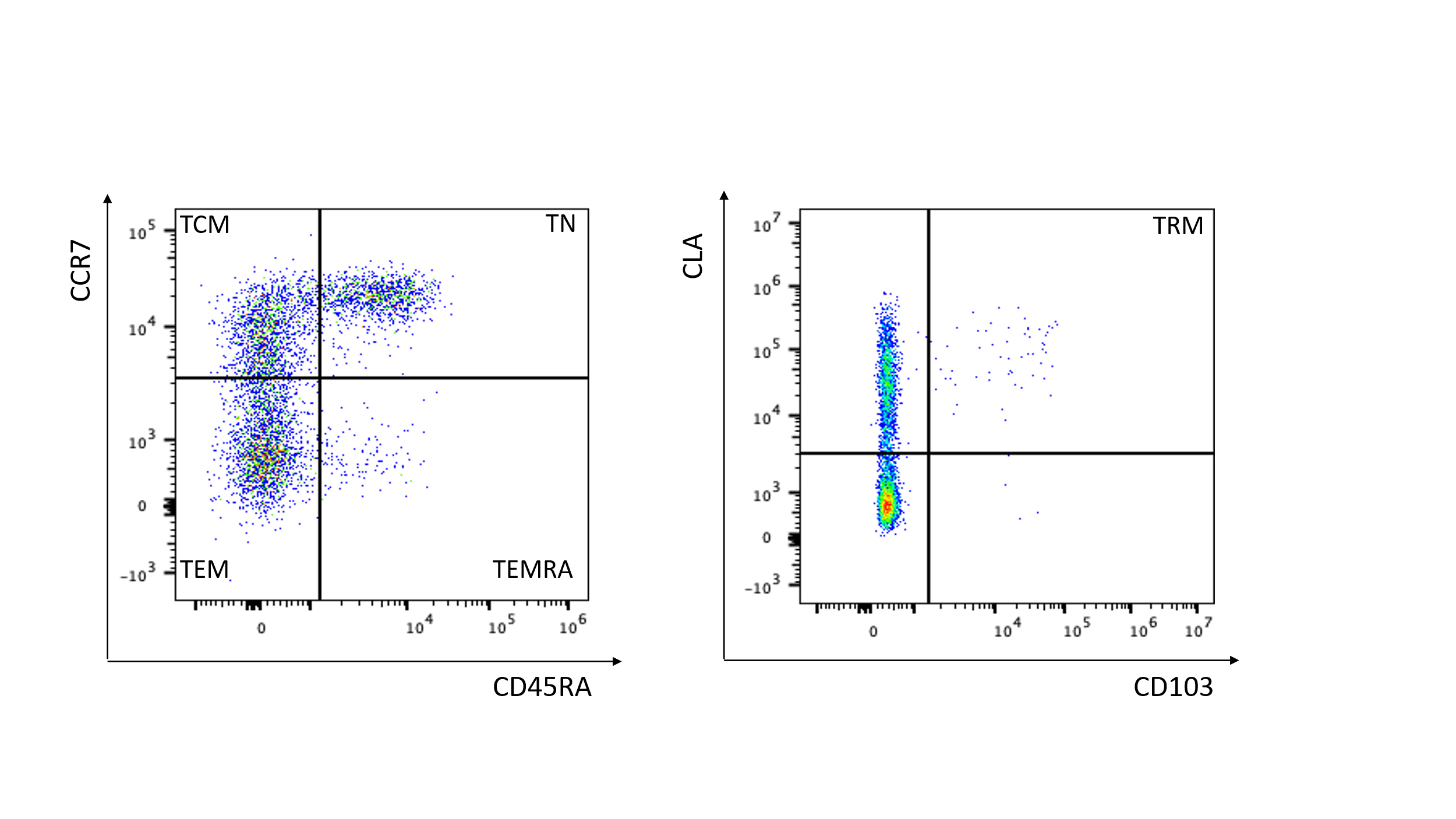


**Suppl. Fig. 3.** **Analysis of CD4+ and CD8+ T cell subsets by multicolor flow cytometry**. CD4+ and CD8+ T cells were further stratified based on CD45RA and CCR7 expression to delineate four major subsets: CD45RA+CCR7+ naïve T cells (TN), CD45RA+CCR7- effector memory T cells re-expressing CD45RA T cells (TEMRA), CD45RA-CCR7+ central memory T cells (TCM) and CD45RA-CCR7- effector memory T cells (TEM) (left). Additionally, CD4+ and CD8+ T cells were subdivided by cutaneous lymphocyte antigen (CLA) and CD103 expression to identify long-lived tissue-resident memory T cells (TRM), characterized as CLA⁺CD103⁺ cells (right).


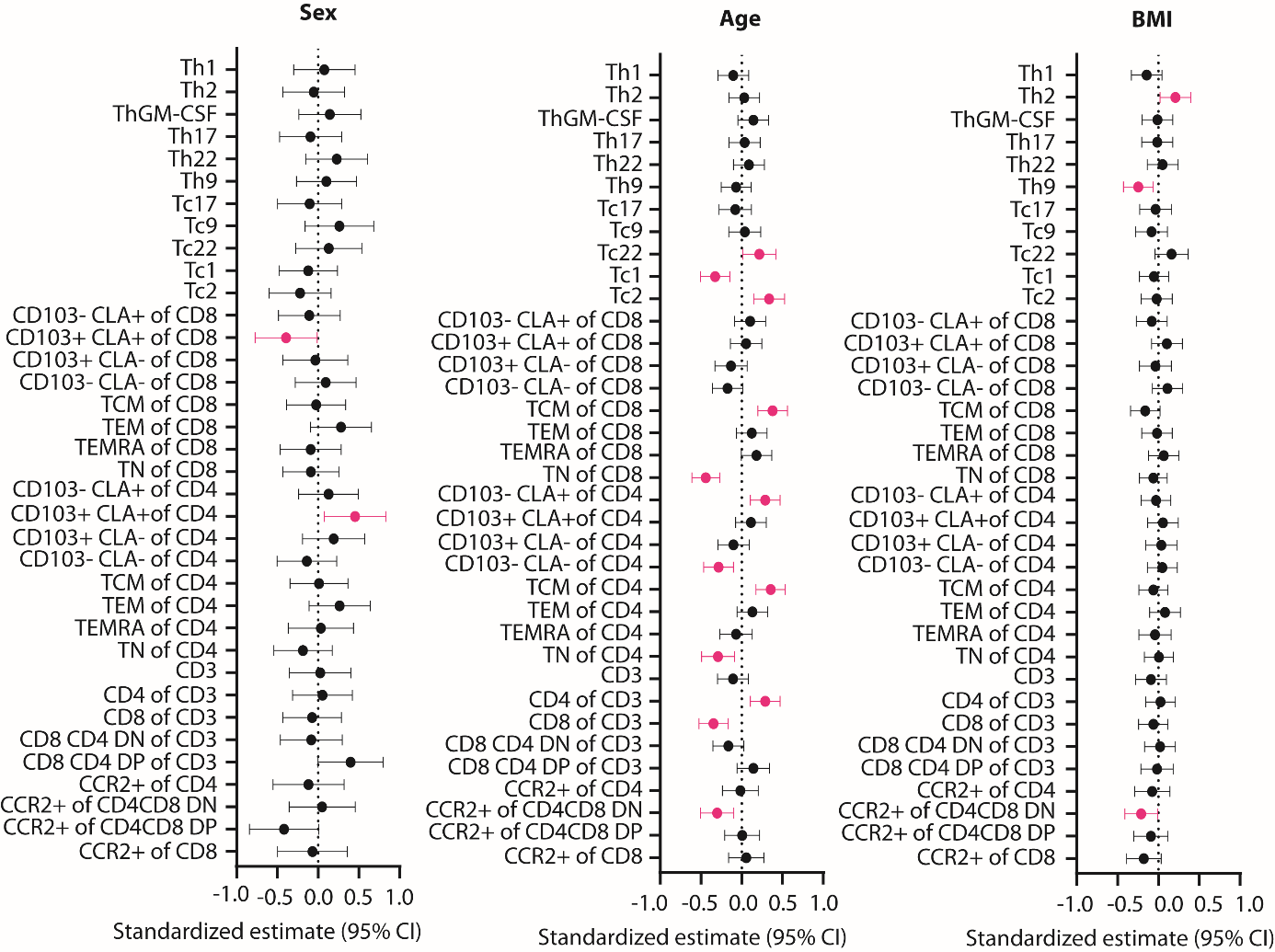


**Suppl. Fig. 4. Individual T cell subsets were influenced by age, sex, and BMI**. Frequencies of T cell subsets were normalized and analyzed for the confounding effects of sex, age, and body mass index (BMI) using linear regression. The standardized estimates and 95% confidence intervals (CI) are illustrated in the forest plots, with significant results highlighted in pink.

|  | **Subclinical psoriatic arthritis**  **(n=8)** | | **Psoriatic arthritis**  **(n=62)** | | **Statistic** |
| --- | --- | --- | --- | --- | --- |
|  | **n** | **%** | **n** | **%** | **p-value** |
| **No joint tenderness & arthralgia** | 3 | 37.5 | 0 | 0.0 | **0.0010** |
| **Joint tenderness & arthralgia** | 5 | 62.5 | 62 | 100.0 |  |
| **No arthritis** | 8 | 100.0 | 15 | 24.1 | **0.0004** |
| **Monarthritis** | 0 | 0.00 | 4 | 6.5 |  |
| **Oligoarthritis** | 0 | 0.00 | 14 | 22.6 |  |
| **Polyarthritis** | 0 | 0.00 | 29 | 46.8 |  |
|  |  |  |  |  |  |
| **Clinically diagnosed enthesitis** | 0 | 0.00 | 41 | 66.1 | **0.0005** |
|  |  |  |  |  |  |
| **Axial disease manifestation** | 0 | 0.00 | 10 | 16.1 | 0.5909 |
|  |  |  |  |  |  |
| **Dactylitis** | 0 | 0.0 | 11 | 17.7 | 0.3400 |
|  |  |  |  |  |  |
| **Uveitis** | 0 | 0.0 | 2 | 3.2 | 1.000 |
|  |  |  |  |  |  |
|  | **M** | **SD** | **M** | **SD** | **Mann-Whitney *U* test** |
| **HAQ** | 0.9 | 0.4 | 0.9 | 0.8 | 0.8968 |
| **Tjc** | 4.0 | 6.0 | 13.9 | 14.8 | 0.0509 |
| **Sjc** | 0.3 | 0.5 | 6.0 | 9.6 | **0.0013** |

**Suppl. Table 2.** **Clinical characterization of patients with subclinical psoriatic arthritis and psoriatic arthritis.** Data are presented as mean (M) ± standard deviation (SD) or frequencies as appropriate. Frequencies were compared using Fisher’s exact test. Data analysis was performed using Mann-Whitney U test for non-parametric data. *Abbreviations: HAQ – Health Assessment Questionnaire, M – mean, n – number, SD – standard deviation, sjc - swollen joint count, tjc – tender joint count.*


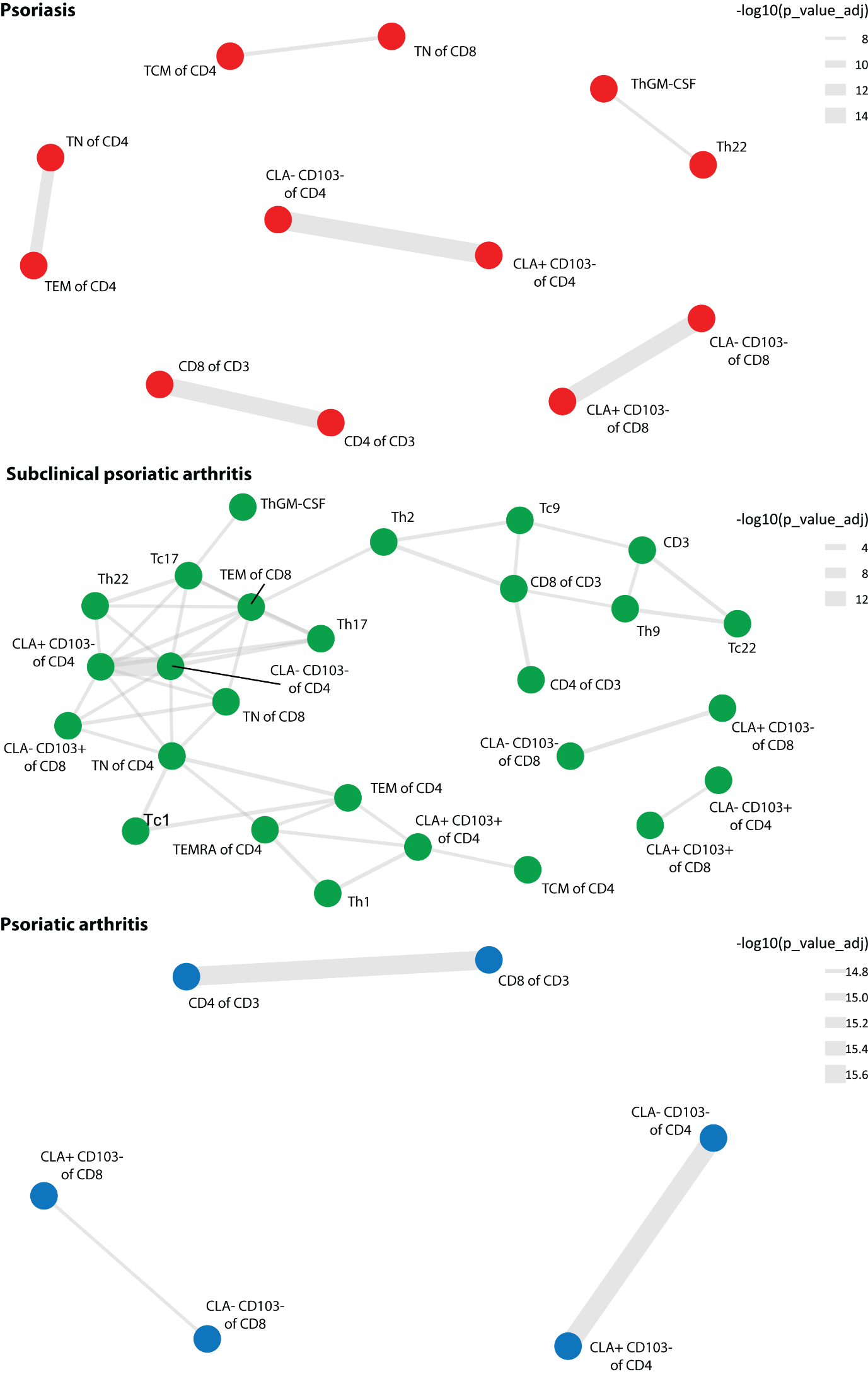


**Suppl. Fig. 5.** **Patients with subclinical psoriatic arthritis had a more intricate and interconnected T cell subset network compared to patients with psoriasis and psoriatic arthritis**. The interrelationships between the T cell subsets within each group are illustrated by correlation networks. Correlations with Spearman’s r correlation coefficient > 0.7 are indicated by gray lines between colored edges, which represent the T cell subsets. The network is visualized with edge widths scaled by the -log10 of adjusted p-values (False Discovery rate Benjamini-Hochberg) according to Fruchterman-Reingold-Layout algorithm.


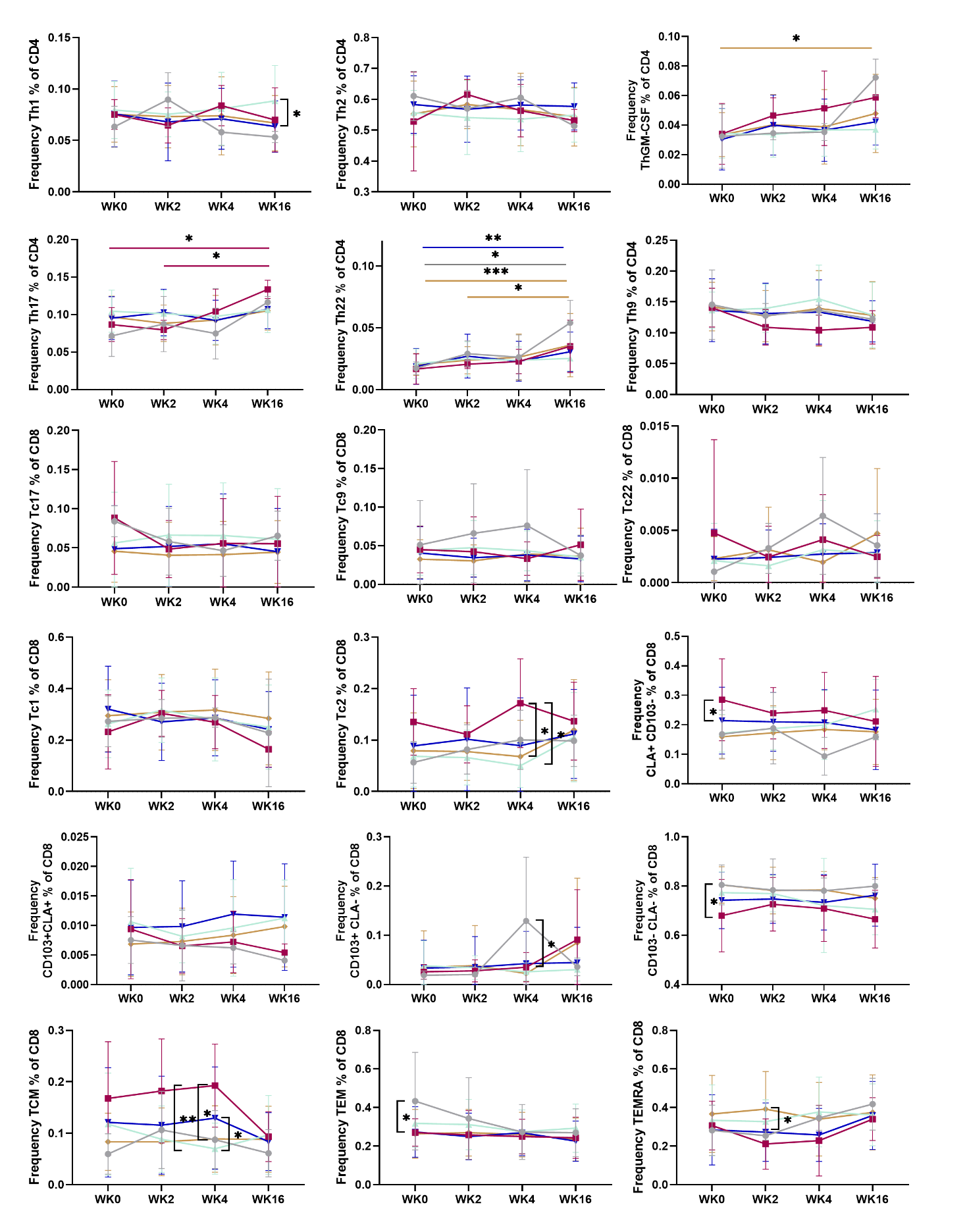


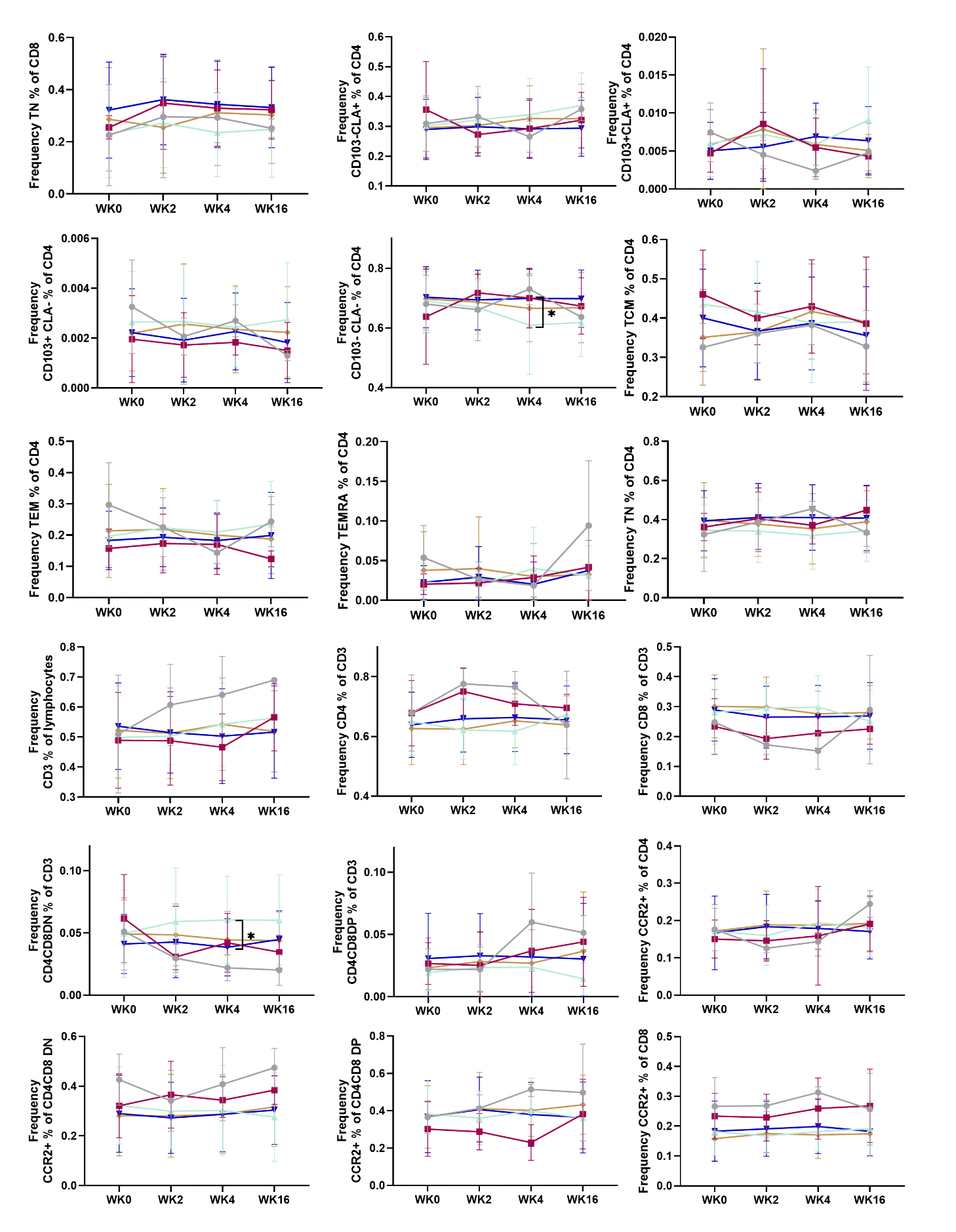


**Suppl. Fig. 6.** **The effect of csDMARDs, tsDMARDs, and biologics on the frequency of specific T cell subsets is relatively small in peripheral blood.** To assess therapeutic effects on T cell subset frequencies, 114 patients underwent immunophenotyping at therapy initiation and after 2, 4, and 16 weeks. The investigated patients received therapy initiation with csDMARD [n=5] (grey), tsDMARD [n=8] (red) or biologic therapy (TNF inhibitor [n=26] (light green), IL-17(R) inhibitors [n=42] (blue) or IL-(12)/23 inhibitors [n=33] (ocher)). The frequency of T cells was compared on the one hand between different timepoints and on the other hand between the respective therapies at each timepoint using Two-Way ANOVA with Tukey’s post hoc test. The statistic results are depicted in the respective graphs indicated by stars (*p<0.050, **p<0.0021, ***p<0.0002, ****p<0.0001).

**
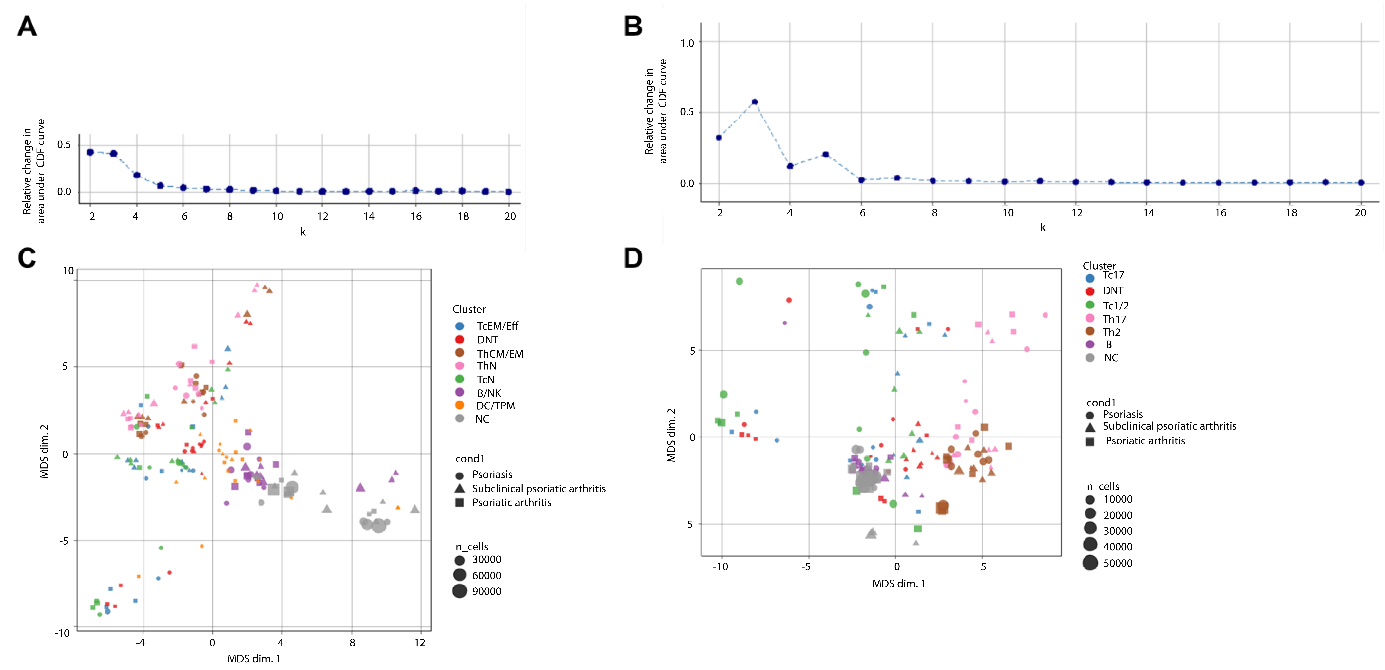
**

**Suppl. Fig. 7. Quality control plots of FlowSOM clustering**. **A)** Delta-area plot for panel 1 suggesting a maximum metacluster number of 8. **B)** Delta-area plot for panel 2 suggesting a maximum metacluster number of 7. **C)** Multi-dimensional scaling (MDS) plot showing adequate clustering for panel 1 and **D)** for panel 2.

**
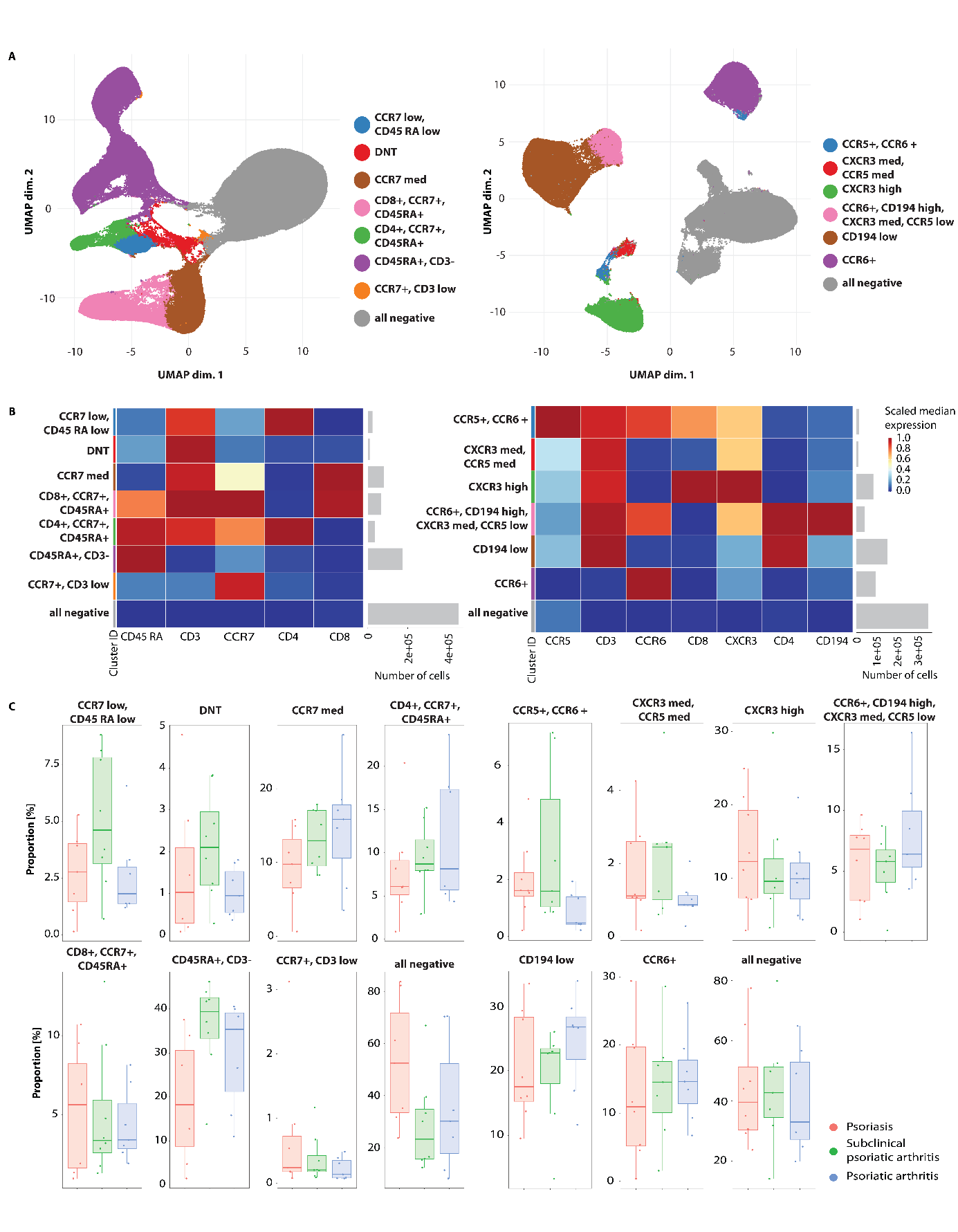
**

**Suppl. Fig. 8.** **ML-based comparison of T cell subsets identified by ML in patients with psoriasis, subclinical psoriatic arthritis and psoriatic arthritis. A)** FlowSOM-clustering color-coded on a UMAP representation of dataset 1 (left) and dataset 2 (right) reveals ML-based identification of distinct T cell subtypes. **B)** Heatmaps illustrate metaclusters characteristics and abundances from dataset 1 (left) and dataset 2 (right). **C)** Box plots show differential regulation of metaclusters in the subclinical psoriatic arthritis group.

| **Dataset** | **Cluster** | **Characteristics** | **Estimated cell subsets** | |
| --- | --- | --- | --- | --- |
|  |  |  | **Short Name** | **Long Name** |
| 1 | 1 | CD4+, CCR7+, CD45RA+ | TN CD4+ | Naive CD4 T cells |
| 1 | 2 | CCR7 med | TCM/TEM CD4+ | Central Memory/Effector Memory CD4 T cells |
| 1 | 3 | CD8+, CCR7+, CD45RA+ | TN CD8+ | Naive CD8 T cells |
| 1 | 4 | CCR7 low, CD45RA low | TEM CD8+ | Effector Memory/Effector CD8 T cells |
| 1 | 5 | Double Negative | DN | Double Negative T cells |
| 1 | 6 | CD45RA+, CD3-negative | B/NK | Naive B Cells/NK cells |
| 1 | 7 | CCR7+, CD3low | DC/TPM | Dendritic Cells/Immature T cells |
| 1 | 8 | All Negative (NK cells, DCs, debris) | NC | Non-T cell populations |
| 2 | 1 | CCR6+, CD194high, CXCR3med, CCR5low | Th17 | T-helper 17 cells |
| 2 | 2 | CD194low | Th2 | T-helper 2 cells |
| 2 | 3 | CCR5+, CCR6+ | Tc17 | T-cytotoxic 17 cells |
| 2 | 4 | CXCR3 high | Tc1/2 | T-cytotoxic 1/2 cells |
| 2 | 5 | CXCR3med, CCR5med | DN | Double Negative T cells |
| 2 | 6 | CCR6+ | B | B cells |
| 2 | 7 | All Negative (NK cells, DCs, debris) | NC | Non-classifiable |

**Suppl. Table 3.** **Naming of metaclusters and the respective rationale**. The table provides a rationale for the metacluster naming conventions, rooted in the observed marker expressions and the biological characteristics of each subset, as identified by the machine learning analysis.

**References**

1. Nast A, Altenburg A, Augustin M, Boehncke W, Härle P, Klaus J, et al. Deutsche S3‐Leitlinie zur Therapie der Psoriasis vulgaris, adaptiert von EuroGuiDerm – Teil 2: Therapiemonitoring, besondere klinische Situationen und Komorbidität. J Deutsche Derma Gesell. 2021 July;19(7):1092–117.

2. Scher JU, Ogdie A, Merola JF, Ritchlin C. Preventing psoriatic arthritis: focusing on patients with psoriasis at increased risk of transition. Nat Rev Rheumatol. 2019 Mar;15(3):153–66.

3. Perez-Chada LM, Haberman RH, Chandran V, Rosen CF, Ritchlin C, Eder L, et al. Consensus terminology for preclinical phases of psoriatic arthritis for use in research studies: results from a Delphi consensus study. Nat Rev Rheumatol. 2021 Apr;17(4):238–43.

4. Zabotti A, De Marco G, Gossec L, Baraliakos X, Aletaha D, Iagnocco A, et al. EULAR points to consider for the definition of clinical and imaging features suspicious for progression from psoriasis to psoriatic arthritis. Annals of the Rheumatic Diseases. 2023 Sept;82(9):1162–70.

5. De Marco G, Zabotti A, Baraliakos X, Iagnocco A, Aletaha D, Gisondi P, et al. Characterisation of prodromal and very early psoriatic arthritis: a systematic literature review informing a EULAR taskforce. RMD Open. 2023 June;9(2):e003143.

6. Taylor W, Gladman D, Helliwell P, Marchesoni A, Mease P, Mielants H, et al. Classification criteria for psoriatic arthritis: Development of new criteria from a large international study. Arthritis Rheum. 2006 Aug;54(8):2665–73.

7. Gossec L, Kerschbaumer A, Ferreira RJO, Aletaha D, Baraliakos X, Bertheussen H, et al. EULAR recommendations for the management of psoriatic arthritis with pharmacological therapies: 2023 update. Ann Rheum Dis. 2024 Mar 18;ard-2024-225531.

8. Coates LC, Soriano ER, Corp N, Bertheussen H, Callis Duffin K, Campanholo CB, et al. Group for Research and Assessment of Psoriasis and Psoriatic Arthritis (GRAPPA): updated treatment recommendations for psoriatic arthritis 2021. Nat Rev Rheumatol. 2022 Aug;18(8):465–79.

9. Callis Duffin K, Gottlieb AB. Outcome Measures for Psoriasis Severity: A Report from the GRAPPA 2012 Annual Meeting. J Rheumatol. 2013 Aug;40(8):1423–4.

10. Schoels M, Knevel R, Aletaha D, Bijlsma JWJ, Breedveld FC, Boumpas DT, et al. Evidence for treating rheumatoid arthritis to target: results of a systematic literature search. Annals of the Rheumatic Diseases. 2010 Apr 1;69(4):638–43.

11. Härle P, Hartung W, Lehmann P, Ehrenstein B, Schneider N, Müller H, et al. GEPARD-Patientenfragebogen: Erfassung von Patienten mit Psoriasisarthritis aus einem ambulanten dermatologischen Patientenpool. Z Rheumatol. 2010 Mar;69(2):157–63.

12. Härle P, Letschert K, Wittig B, Mrowietz U. Sensitivity of the GEPARD Patient Questionnaire to Identify Psoriatic Arthritis in Patients with Psoriasis in Daily Practice: The GEPARD-Life Study. Dermatology. 2016;232(5):597–605.

13. Mease PJ, Gladman DD, Helliwell P, Khraishi MM, Fuiman J, Bananis E, et al. Comparative performance of psoriatic arthritis screening tools in patients with psoriasis in European/North American dermatology clinics. Journal of the American Academy of Dermatology. 2014 Oct;71(4):649–55.

14. Fodor D, Rodriguez-Garcia SC, Cantisani V, Hammer HB, Hartung W, Klauser A, et al. The EFSUMB Guidelines and Recommendations for Musculoskeletal Ultrasound – Part I: Extraarticular Pathologies. Ultraschall Med. 2022 Feb;43(01):34–57.

15. Bruyn GA, Iagnocco A, Naredo E, Balint PV, Gutierrez M, Hammer HB, et al. OMERACT Definitions for Ultrasonographic Pathologies and Elementary Lesions of Rheumatic Disorders 15 Years On. J Rheumatol. 2019 Oct;46(10):1388–93.

16. Husted JA, Gladman DD, Long JA, Farewell VT. A modified version of the Health Assessment Questionnaire (HAQ) for psoriatic arthritis. Clin Exp Rheumatol. 1995;13(4):439–43.

17. Mousset CM, Hobo W, Woestenenk R, Preijers F, Dolstra H, van der Waart AB. Comprehensive Phenotyping of T Cells Using Flow Cytometry. Cytometry Pt A. 2019 June;95(6):647–54.

18. Staser KW, Eades W, Choi J, Karpova D, DiPersio JF. OMIP‐042: 21‐color flow cytometry to comprehensively immunophenotype major lymphocyte and myeloid subsets in human peripheral blood. Cytometry Pt A. 2018 Feb;93(2):186–9.

19. Hipp AV, Bengsch B, Globig AM. Friend or Foe - Tc17 cell generation and current evidence for their importance in human disease. Discov Immunol. 2023;2(1):kyad010.

20. Parween F, Singh SP, Kathuria N, Zhang HH, Ashida S, Otaizo-Carrasquero FA, et al. Migration arrest and transendothelial trafficking of human pathogenic-like Th17 cells are mediated by differentially positioned chemokines. Nat Commun. 2025 Feb 26;16(1):1978.

21. Klicznik MM, Morawski PA, Höllbacher B, Varkhande SR, Motley SJ, Kuri-Cervantes L, et al. Human CD4^+^ CD103^+^ cutaneous resident memory T cells are found in the circulation of healthy individuals. Sci Immunol. 2019 July 5;4(37):eaav8995.
